# Machine learning prediction of anxiety symptoms in social anxiety disorder: utilizing multimodal data from virtual reality sessions

**DOI:** 10.3389/fpsyt.2024.1504190

**Published:** 2025-01-07

**Authors:** Jin-Hyun Park, Yu-Bin Shin, Dooyoung Jung, Ji-Won Hur, Seung Pil Pack, Heon-Jeong Lee, Hwamin Lee, Chul-Hyun Cho

**Affiliations:** ^1^ Department of Biomedical Informatics, Korea University College of Medicine, Seoul, Republic of Korea; ^2^ Department of Psychiatry, Korea University College of Medicine, Seoul, Republic of Korea; ^3^ Graduate School of Health Science and Technology, Department of Biomedical Engineering, Ulsan National Institute of Science and Technology (UNIST), Ulsan, Republic of Korea; ^4^ School of Psychiatry, Korea University, Seoul, Republic of Korea; ^5^ Department of Biotechnology and Bioinformatics, Korea University, Sejong, Republic of Korea

**Keywords:** machine learning, multimodal data, digital phenotyping, digital psychiatry, social anxiety disorder, virtual reality intervention, anxiety prediction

## Abstract

**Introduction:**

Machine learning (ML) is an effective tool for predicting mental states and is a key technology in digital psychiatry. This study aimed to develop ML algorithms to predict the upper tertile group of various anxiety symptoms based on multimodal data from virtual reality (VR) therapy sessions for social anxiety disorder (SAD) patients and to evaluate their predictive performance across each data type.

**Methods:**

This study included 32 SAD-diagnosed individuals, and finalized a dataset of 132 samples from 25 participants. It utilized multimodal (physiological and acoustic) data from VR sessions to simulate social anxiety scenarios. This study employed extended Geneva minimalistic acoustic parameter set for acoustic feature extraction and extracted statistical attributes from time series-based physiological responses. We developed ML models that predict the upper tertile group for various anxiety symptoms in SAD using Random Forest, extreme gradient boosting (XGBoost), light gradient boosting machine (LightGBM), and categorical boosting (CatBoost) models. The best parameters were explored through grid search or random search, and the models were validated using stratified cross-validation and leave-one-out cross-validation.

**Results:**

The CatBoost, using multimodal features, exhibited high performance, particularly for the Social Phobia Scale with an area under the receiver operating characteristics curve (AUROC) of 0.852. It also showed strong performance in predicting cognitive symptoms, with the highest AUROC of 0.866 for the Post-Event Rumination Scale. For generalized anxiety, the LightGBM’s prediction for the State-Trait Anxiety Inventory-trait led to an AUROC of 0.819. In the same analysis, models using only physiological features had AUROCs of 0.626, 0.744, and 0.671, whereas models using only acoustic features had AUROCs of 0.788, 0.823, and 0.754.

**Conclusions:**

This study showed that a ML algorithm using integrated multimodal data can predict upper tertile anxiety symptoms in patients with SAD with higher performance than acoustic or physiological data obtained during a VR session. The results of this study can be used as evidence for personalized VR sessions and to demonstrate the strength of the clinical use of multimodal data.

## 1 Introduction

Social anxiety disorder (SAD) is characterized by an excessive fear of negative evaluation or distorted cognitive perception triggered by social or performance situations (1). SAD is one of the most common mental disorders in the general population, with an estimated lifetime prevalence of up to 12% in the US (2). Therefore, considerable effort has been devoted to the development of therapeutic approaches for SAD. Currently, the combination of cognitive behavioral therapy (CBT) and antidepressant medication with carefully planned procedures is considered the gold standard treatment for SAD (3, 4). However, with advances in science and technology, virtual reality (VR) has accelerated a paradigm shift in psychiatric treatment (5). In particular, given the nature of VR technology, which makes it possible to mimic real-life social interactions within a therapeutic context, CBT with virtual exposure to feared stimuli has been assumed to be a promising alternative to current practice in managing patients with SAD (6, 7).

From the current perspective, early, accurate, and objective assessment of mental states, as well as prompt therapeutic management, is regarded as the most effective way to improve disease prognosis (8). Concurrently, machine learning (ML) technology is used to develop prediction, classification, and therapeutic solutions for mental states, making precision medicine a reality (9, 10). Therefore, ML technology has been incorporated into VR exposure therapy (VRET) to treat SAD (11, 12). In support of this, considerable effort has been devoted to developing an ML-based prediction of individuals’ mental states in real time for exposure therapy in virtuo using central and peripheral biosignals (13–15). Specifically, biofeedback framework, defined as the process of teaching patients to intentionally regulate their physiological response for improving mental states (e.g., decreased stress or anxiety) through VR-embedded visual feedback (e.g., growing tree branches or gently moving particles), has been combined with VRET and ML technology (16). However, given the capability of ML to process multimodal datasets, there is still room for improvement to provide more robust interventions for patients with SAD (17–20). From a neuroscientific perspective, a multi-modality approach, which involve fusing and analyzing different types of data, including medical images (e.g., magnetic resonance images (MRI) and structural MRI (sMRI)), physiological signals (e.g., electrocardiogram, electromyogram, and electroencephalogram), acoustic features, and speech transcript, provides a fuller understanding of mental conditions (21). For example, multimodal feature sets via a combination of different biomarkers, such as sMRI, fluorodeoxyglucose positron emission tomography (FDG-PET), cerebrospinal fluid performed up to 6.7% better than unimodal features in classifying patients with Alzheimer’s disease from healthy controls (22). Similarly, recent study demonstrated the potential of ML-enabled detection of neurotypical and attention-deficit/hyperactivity disorder populations by incorporating multimodal physiological data, including electrodermal activity, heart rate variability, and skin temperature (23). Therefore, in this study, the predictive performance of ML models utilizing multimodal data from VRET sessions was evaluated based on their medical applicability in personalized therapy.

When implementing CBT for SAD, it is important to recognize that SAD is characterized by various symptoms, including heightened social anxiety/fear, distorted self-referential attention/rumination, and maladaptive beliefs (fear of negative evaluation, humiliation, and embarrassment) (24–26). Empirical research has indicated heterogeneity in treatment responses among patients with anxiety disorders over therapy sessions (27–29). For example, patients may show early or delayed recovery and a steady or moderate decline in symptoms (30, 31). Moreover, patients may exhibit attenuated or steep slopes in their symptom trajectory (32). Furthermore, symptom variability has been observed in patients with SAD (33). Therefore, examining a broad array of symptoms throughout CBT is crucial for identifying whether the treatment works and how much progress has been made. Thus, in this study, a comprehensive assessment battery was administered to participants, and their SAD symptom responses during VRET were predicted using an ML approach to provide information on the trajectory of session-to-session changes in the symptom facets. Such an approach could help deliver tailored interventions for heterogeneous patients, identify those who may be at risk of not responding, and contribute to therapists’ evidence-based clinical decision making.

This study aimed to build predictive models of upper tertile symptoms related to SAD using machine learning algorithms by utilizing acoustic and physiological features, as well as combined multimodal data from VRET sessions, and to evaluate the effectiveness of these predictive models.

## 2 Materials and methods

### 2.1 Participants

A total of 32 young adults were recruited through internet advertisements. Participants with SAD were eligible if they met the Diagnostic and Statistical Manual of Mental Disorders, Fifth Edition criteria for SAD, which was assessed using the Mini-International Neuropsychiatric Interview (34), and if they had a score ≥ 82 on the Korean version of the Social Avoidance and Distress Scale (35). The exclusion criteria for all participants were (1) having a lifetime or current mental illness or neurological disorder that might elicit severe side effects from a VR experience [e.g., schizophrenia spectrum disorder, bipolar disorder, posttraumatic stress disorder, panic disorder, substance use disorders, autism spectrum disorder [ASD], epilepsy, traumatic brain injury, and suicide attempts) (2); having an intellectual disability (IQ < 70; estimated using the short version of the Korean Wechsler Adult Intelligence Test Fourth Edition (36)]; and (3) receiving psychotropic medication or psychotherapy at the time of research enrollment.

Of the initial 32 participants, data from 7 individuals were omitted from the analysis because of sensor malfunctions. Thus, physiological and acoustic data were derived from 4 sessions of 25 individuals, resulting in 100 samples. In addition, participants were allowed to repeat VR exposure scenarios at their request for extra training, resulting in 89 additional samples. After removing 57 samples, which were considered outliers due to errors in audio recordings, samples where no speech was made, and instances where time-series data contained values like -1 exceeding 30%, we finally obtained 132 samples. Consequently, the final dataset for the ML analysis consisted of 132 samples, expanded by incorporating additional data obtained from extra sessions, which comprised both multimodal data and clinical and psychological scale values collected from 25 participants. All procedures in this study were performed in accordance with the guidelines of Declaration of Helsinki regarding the ethical principles for medical research involving human participants. This study was approved by the Institutional Review Board of the Korea University Anam Hospital (IRB no. 2018AN0377). All participants provided written informed consent.

### 2.2 VR sessions for SAD

The VR intervention was designed to immerse participants in scenarios that simulated social anxiety within contexts pertinent to SAD therapy, aiming to facilitate the confrontation and mitigation of their fear. The intervention consisted of six VR sessions, each structured into three phases: introductory, main, and concluding. These sessions were categorized into three difficulty tiers (easy, medium, and hard), based on the challenges presented during the main phase. The initial phase acquainted participants with the virtual setting and employed meditation-based relaxation exercises. The main phase was initiated by introducing seven to eight virtual characters, simulating an interaction scenario akin to the first day of college class. Participants began their self-introduction by activating the recording function using an icon on the head-mounted display (HMD). During this phase, they could adjust the session’s difficulty by choosing between easy, medium, or hard levels, which influenced the responses of the virtual characters. The concluding phase mirrored the introductory phase, offering a meditation-based VR experience to soothe participants’ minds. Initially, all participants engaged at an easy level. Starting from the second session, they were given the autonomy to select their preferred difficulty level, allowing for adjustment of the challenge to suit their individual preferences, thereby ensuring a personalized therapeutic experience. Additional details concerning the intervention can be found in a study by Kim et al. (37). The sample of the VR sessions used in this intervention can be found at Youtube^1^.

### 2.3 Measures

**Figure 1:**
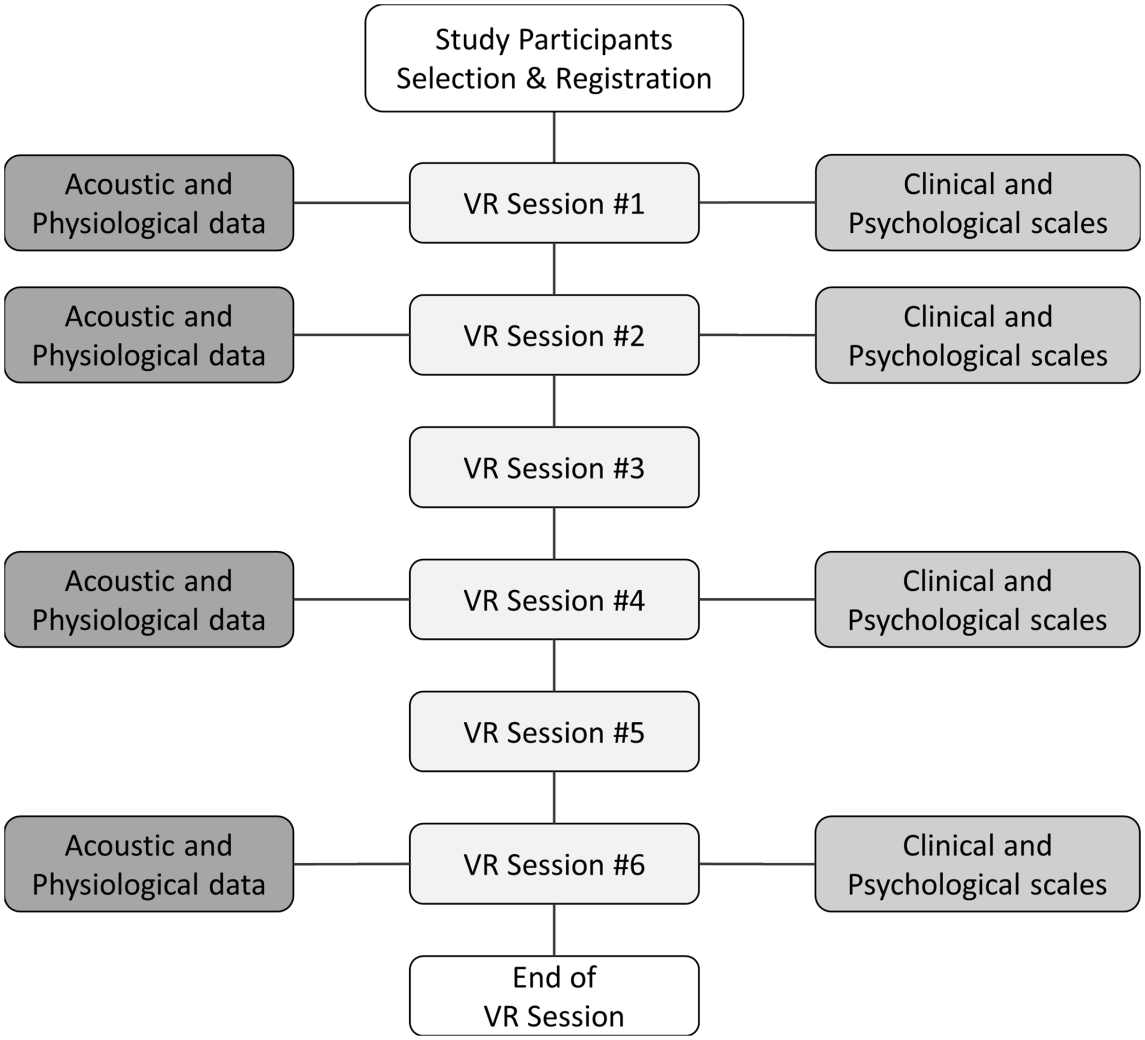
Overview of data collection during VR sessions. VR, virtual reality. This figure shows the overall process of data extraction.

During the main phase of each VR session, participants were subjected to *in situ* measurements of video recordings and autonomic physiological data. Note that analyses include data gathered only from the main phase in which social interaction between the user and virtual avatars took place. **Figure 1** provides a comprehensive description of the data-collection methodology. Heart rate (HR) and galvanic skin response (GSR) were measured to assess physiological responses during speech because of their close relationship with anxiety (38–40). Using a Shimmer3 GSR+ with three channels, we measured the skin conductance on the index and middle fingers of the non-dominant hand at 52 Hz and cardiac volume using an earlobe infrared sensor, converting this to HR data. During the VR sessions, the participants’ voices were captured with an HTC Vive HMD microphone for vocal analysis, enhancing the depth of the study.

A comprehensive assessment battery was used to measure the symptom characteristics at the first, second, fourth, and sixth VR sessions. For core symptoms of SAD, we used the Korean versions of the Social Phobia Scale (K-SPS) (41, 42), Liebowitz Social Anxiety Scale (K-LSAS) (43, 44), Social Avoidance and Distress Scale (K-SADS) (35, 45), and Social Interaction Anxiety Scale (K-SIAS) (42, 46). Cognitive symptoms of SAD were assessed using the Post-Event Rumination Scale (PERS) (47, 48), Brief Fear of Negative Evaluation (BFNE) (35, 45) scale, and Internalized Shame Scale (ISS) (49, 50). Regarding generalized anxiety symptoms, the State-Trait Anxiety Inventory (STAI) (51, 52) and Beck Anxiety Inventory (BAI) (53, 54) were evaluated. A detailed description of each assessment is provided in **Table 1**, and we utilized the total scores from each clinical and psychological scale.

**Table 1:** A detailed description of the clinical and psychological scales.

Core Symptom of SAD
Social Phobia Scale (SPS)
The SPS was designed to assess the fear of being scrutinized during activities and performance tasks. It consists of 20 items and each answer is scored on a scale of 0 (not at all) to 4 (extremely). Total scores range from 0 to 80, with higher scores representing greater anxiety about being observed. The Korean version of the SPS was used.
Liebowitz Social Anxiety Scale (LSAS)
The LSAS was designed to assess the fear or anxiety and avoidance of various social interaction and performance situations. It consists of 24 items on two separate scales, assessing fear or anxiety (ranging from 0 = none, to 3 = severe) and avoidance (ranging from 0 = never to 3 = usually). Higher total scores indicate more severe social anxiety symptoms. The Korean version of the SPS was used.
Social Avoidance and Distress Scale (SADS)
The SADS was designed to assess distress in social situations and the avoidance tendency in social interactions. It consists of 28 items on a true-false scale. In the Korean version of the SADS, each item was assessed on a 5-point scale. Higher total scores indicate more severe social anxiety symptoms.
Social Interaction Anxiety Scale (SIAS)
The SIAS was designed to assess anxiety in social interactional situations. It consists of 20 items and each answer is scored on a scale of 0 (not at all) to 4 (extremely). Total scores range from 0 to 80, with higher scores representing greater social interaction anxiety. The Korean version of the SIAS was used.
Cognitive Symptom of SAD maintenance
Post-Event Rumination Scale (PERS)
The PERS was designed to assess the frequency of post-event ruminations in social situations. It comprises two scales including negative rumination (15 items) and positive rumination (9 items). Each answer is scored on a scale of 0 (never) to 4 (very often); higher scores indicate more frequent rumination.
Brief Fear of Negative Evaluation (BFNE)
The BFNE is a 12-item version of the original 30-item fear of negative evaluation scale and measures the degree of fear or worry of negative evaluation by others. The Korean version was used in this study. Each item was scored on a 5-point Likert-type scale ranging from 1 (strongly disagree) to 5 (strongly agree), and scores were summed with higher scores reflecting greater levels of anxiety or fear.
Internalized Shame Scale (ISS)
The ISS was designed to assess trait-shame or internalized shame. It consists of a 24-item shame scale and a 6-item self-esteem scale in which each answer is scored on a scale of 0 (not at all) to 4 (extremely). Total scores range from 0 to 120, with higher scores representing higher level of trait-shame.
Generalized Anxiety
State-Trait Anxiety Inventory (STAI)
The STAI was designed to assess the level of state and trait anxiety. It consists of a 20-state anxiety scale (STAI-State) and a 20-trait anxiety scale (STAI-Trait). Both scales range from 1 (almost never) to 4 (almost always) higher scores indicating a higher level of state-trait anxiety.
Beck Anxiety Inventory (BAI)
The BAI was designed to assess the intensity of somatic (hands trembling, face flushed, heart pounding) and cognitive (feeling terrified, fearing the worst, fear of losing control, fear of dying) anxiety symptoms. It consists of 21 items and each answer is scored on a scale of 0 (not at all) to 3 (severely). Total scores range from 0 to 63, with higher scores representing more severe symptoms. As evaluating anxiety symptoms in one-week time frame, the BAI is considered as a measure of state rather than trait anxiety. The Korean version of the BAI was used.

### 2.4 Data preprocessing

#### 2.4.1 Labeling procedure with clinical and psychological scales

Scores from the 132 samples were divided into tertiles for each clinical and psychological scale (K-SPS, K-LSAS, K-SADS, K-SIAS, PERS, BFNE, ISS, STAI-State, STAI-Trait, and BAI), resulting in three classification groups per scale. Then, the top tertile for each scale was grouped into a “severe group,” and the remaining samples formed a “non-severe group,” using the severe group labels as the ground truth for machine learning prediction.

#### 2.4.2 Acoustic features extraction process

Video recordings of VR sessions were converted to waveform audio file format (WAV) format for analysis. Following the removal of samples with errors in audio recordings, samples where no speech was made, and samples containing outliers in physiological data, we obtained a total of 132 WAV files for machine learning training. From each of these files, we extracted a total of 88 acoustic features included in the extended Geneva Minimalistic Acoustic Parameter Set (eGeMAPS) (55). **Supplementary Table S1** details the acoustic features analyzed using eGeMAPS. The features were broadly categorized into frequency-related metrics, energy dynamics, spectral properties, and temporal patterns, and all 88 features were extracted using the openSMILE toolkit (56).

#### 2.4.3 Physiological features extraction process

The collected HR and GSR time series data were aligned with the length of the voice recordings. Samples with excessive negative readings were removed, considered outliers such as instances where the proportion of -1 values exceeded 30%. Among the 132 usable samples, missing values in HR and GSR were imputed using forward and backward imputation techniques (57). Further data cleansing was achieved by applying the interquartile range (IQR) technique (58), which was chosen to manage the variability in HR and GSR data. The IQR method is effective for reducing noise caused by external factors such as sensor misplacement, environmental changes, and user movements, which can lead to abrupt fluctuations. By removing these noise-induced outliers, the IQR technique helps to clarify the essential patterns in the data while maintaining the central tendency, thereby enhancing the reliability of subsequent model training. Following the establishment of a cleaned dataset, a comprehensive suite of 12 statistical features was extracted from both the HR and GSR signals. These features, including the mean, standard deviation, minimum, maximum, mean difference, and maximum difference were calculated to capture the dynamic nature of physiological responses. A detailed description of these features is presented in **Supplementary Table S2**.

### 2.5 Machie learning modeling

In this study, we employed machine learning models including Random Forest (59), eXtreme Gradient Boosting (XGBoost) (60), Light Gradient Boosting Machine (LightGBM) (61), and CatBoost (62) to compare the performance in predicting the severe group for each clinical and psychological scale. These models were implemented in Python version 3.11.5, utilizing the Scikit-learn library version 1.4.0 for classification tasks.

We evaluated the classification models using the stratified k-fold cross-validation with five splits to enhance the model robustness and reduce bias by preserving the proportion of classes across each fold. We employed both grid search and random search methodologies to optimize hyperparameters for the Random Forest, XGBoost, LightGBM, and CatBoost classifiers. This approach ensured alignment with the unique characteristics of our dataset and enhanced predictive accuracy. The range of hyperparameters tuning explored was presented in **Supplementary Table S3**; we extracted the best parameters based on the criterion of maximizing the area under the receiver operating characteristic curve (AUROC). To address the limitation posed by the small data size, we further validated the performance using the leave-one-out cross-validation (LOOCV) with the best parameter models derived from both search methods.

To obtain different perspectives on how well the ML models classified the severe group of each clinical and psychological scale, we evaluated the performance of the ML models using different metrics: accuracy, AUROC, F1 score, sensitivity, positive predictive value (PPV), and negative predictive value (NPV). We also compared the AUROC and PPV performance of all models across all clinical and psychological scales based on individual features. Furthermore, we analyzed the factors influencing ML model predictions using SHapley Additive exPlanations (SHAP) (63), which provided interpretability by quantifying the contribution of each feature to the model’s predictions.

### 2.6 Statistical analysis

Statistical analyses were performed using SciPy version 1.11.1. To discern the variations in acoustic and physiological attributes across the three groups, we assessed the normality of the data distribution using the Shapiro-Wilk test and subsequently applied either one-way analysis of variance (ANOVA) or the Kruskal-Wallis test, depending on the normality of the data. Statistical significance was determined using a false discovery rate of 5%.

## 3 Results

### 3.1 Characteristics of participants and clustered groups

The available sample at the time of analysis consisted of 25 young adults aged 19–31 years (mean age = 23.6 and standard deviation = 3.06) and the majority were female (16/25, 64.0%). Their mean education level was 2.64 of college (13–17 years of education). Descriptive statistics on the scores of clinical and psychological scales by clustered groups (higher, middle, and lower thirds) are presented in **Table 2**. The results of a one-way ANOVA or Kruskal-Wallis test between clustered groups in acoustic and physiological variables for every scale are reported in **Supplementary Table S4**. As shown in this table, statistically significant differences were found only in the K-SPS, K-SIAS, and STAI-Trait scale.

**Table 2:** Descriptive statistics on the various anxiety symptoms for SAD by clustered groups (higher, middle, and lower groups).

Symptom	Groups	Middle group count	Lower group count	Higher group mean (SD)	Middle group mean (SD)	Lower group mean (SD)	Higher group threshold	Middle group threshold	Overall mean (SD)
	Higher group count								
K-SPS	45	50	37	42.09(5.56)	25.36(5.55)	9.11(4.64)	35	17	26.51(14.04)
K-LSAS	45	45	42	94.67(17.29)	68.64(7.42)	39.38(11.37)	79	56	68.20(25.81)
K-SADS	51	41	40	115.67(9.45)	99.61(2.31)	86.12(9.01)	106	96	101.73(14.53)
K-SIAS	45	43	44	54.47(6.22)	40.49(3.08)	23.86(6.97)	46	34	39.71(13.83)
PERS	46	45	41	49.52(4.34)	38.09(2.37)	28.98(4.14)	44	35	39.24(9.17)
BFNE	53	36	43	48.47(4.88)	40.17(2.06)	31.37(4.08)	44	37	40.64(8.31)
ISS	46	46	40	61.30(10.43)	40.22(5.17)	22.52(8.82)	50	33	42.20(17.82)
STAI-State	52	45	35	58.35(6.54)	44.71(3.52)	33.83(3.71)	51	40	47.20(11.12)
STAI-Trait	49	42	41	62.39(5.30)	47.88(3.32)	36.24(3.92)	55	43	49.65(11.68)
BAI	47	50	35	22.85(8.35)	6.70(2.34)	1.86(0.88)	12	4	11.17(11.17)

SAD, social anxiety disorder; SD, standard deviation; K-SPS, the Korean version of the social phobia scale; K-LSAS, the Korean version of the liebowitz social anxiety scale; K-SADS, the Korean version of the social avoidance and distress scale; K-SIAS, the Korean version of the social interaction anxiety scale; PERS, the post-event rumination scale; BFNE, the brief fear of negative evaluation; ISS, the internalized shame scale; STAI-State, the state-trait anxiety inventory-state; STAI-Trait, the state-trait anxiety inventory-trait; BAI, the beck anxiety inventory.

This table shows the characteristics of the group data for each clinical and psychological scale score distribution in thirds.

### 3.2 Machine learning prediction of anxiety symptoms

The complete results of the grid search and random search were provided in **Supplementary Tables S5**-**S7**, and **Supplementary Tables S8**-**S10**, respectively. **Tables 3**–**5** presented the best model performances for each clinical and psychological scale across different modalities, achieved through combinations of grid search or random search with stratified cross-validation.

**Table 3:** The predictive performance of the four machine learning models on the severe group for core symptoms of SAD (K-SPS, K-LSAS, K-SADS, and K-SIAS) using the best parameters from grid search or random search combined with stratified cross-validation.

Variable^a^		Physiological Features				Acoustic Features				Multimodal Features^b^			
		K-SPS	K-LSAS	K-SADS	K-SIAS	K-SPS	K-LSAS	K-SADS	K-SIAS	K-SPS	K-LSAS	K-SADS	K-SIAS
RF (Random Forest)	Accuracy	0.666	0.666	0.590	0.652	0.766	0.696	0.636	0.667	0.803	0.741	0.644	0.720
	AUROC	0.577	0.734	**0.618**	0.702	0.783	0.743	0.732	**0.736**	0.831	0.772	0.697	0.788
	F1-score	0.657	0.661	0.585	0.627	0.762	0.696	0.635	0.660	0.800	0.713	0.642	0.706
	Sensitivity	0.666	0.666	0.590	0.652	0.766	0.696	0.636	0.667	0.803	0.741	0.644	0.720
	PPV	0.659	0.664	0.614	0.706	0.765	0.712	0.644	0.669	0.801	0.727	0.658	0.745
	NPV	0.733	0.754	0.694	0.726	0.805	0.799	0.714	0.771	0.847	0.767	0.736	0.792
XGB (XGBoost)	Accuracy	0.651	0.689	0.546	0.585	0.728	0.742	0.697	0.606	0.712	0.765	0.691	0.674
	AUROC	0.576	0.713	0.615	0.603	0.767	**0.799**	0.741	0.630	0.742	**0.843**	0.709	0.721
	F1-score	0.635	0.684	0.537	0.580	0.722	0.740	0.699	0.609	0.710	0.760	0.680	0.674
	Sensitivity	0.651	0.689	0.546	0.585	0.728	0.742	0.697	0.606	0.712	0.765	0.691	0.674
	PPV	0.643	0.684	0.547	0.591	0.730	0.744	0.711	0.641	0.721	0.780	0.697	0.686
	NPV	0.712	0.757	0.648	0.697	0.783	0.809	0.774	0.746	0.797	0.836	0.734	0.774
LGBM (Light GBM)	Accuracy	0.674	0.650	0.576	0.623	0.727	0.711	0.735	0.644	0.758	0.736	0.787	0.689
	AUROC	**0.626**	0.651	0.605	0.637	**0.788**	0.762	0.754	0.669	0.811	0.820	0.800	0.735
	F1-score	0.661	0.647	0.570	0.615	0.724	0.712	0.734	0.636	0.753	0.737	0.783	0.685
	Sensitivity	0.674	0.650	0.576	0.623	0.727	0.711	0.735	0.644	0.758	0.736	0.787	0.689
	PPV	0.665	0.655	0.596	0.632	0.726	0.717	0.745	0.640	0.756	0.754	0.790	0.698
	NPV	0.721	0.744	0.691	0.742	0.789	0.788	0.773	0.726	0.805	0.818	0.816	0.779
CAT (Cat Boost)	Accuracy	0.652	0.667	0.561	0.683	0.735	0.726	0.712	0.659	0.796	0.728	0.750	0.713
	AUROC	0.567	**0.754**	0.608	**0.712**	0.782	0.779	**0.795**	0.724	**0.852**	0.819	**0.822**	**0.808**
	F1-score	0.645	0.665	0.547	0.660	0.730	0.719	0.712	0.649	0.791	0.727	0.748	0.707
	Sensitivity	0.652	0.667	0.561	0.683	0.735	0.726	0.712	0.659	0.796	0.728	0.750	0.713
	PPV	0.650	0.675	0.558	0.670	0.729	0.726	0.723	0.664	0.796	0.747	0.758	0.719
	NPV	0.726	0.757	0.664	0.787	0.785	0.777	0.778	0.760	0.833	0.817	0.792	0.804

SAD, social anxiety disorder; K-SPS, the Korean version of the social phobia scale; K-LSAS, the Korean version of the liebowitz social anxiety scale; K-SADS, the Korean version of the social avoidance and distress scale; K-SIAS, the Korean version of the social interaction anxiety scale; AUROC, area under the receiver operating characteristic; PPV, positive predictive value; NPV, negative predictive value.

^a^The highest AUROC scores for each clinical and psychological scale are highlighted in bold to denote superior model performance.

^b^The combination of physiological and acoustic features.

**Table 4:** The predictive performance of the four machine learning models on the severe group for cognitive symptoms of SAD (PERS, BFNE, and ISS) using the best parameters from grid search or random search combined with stratified cross-validation.

Variable^a^		Physiological Features			Acoustic Features			Multimodal Features^b^		
		PERS	BFNE	ISS	PERS	BFNE	ISS	PERS	BFNE	ISS
RF (Random Forest)	Accuracy	0.689	0.446	0.614	0.643	0.690	0.696	0.726	0.636	0.644
	AUROC	**0.744**	0.397	0.600	0.653	**0.758**	0.669	0.772	0.722	0.629
	F1-score	0.687	0.444	0.614	0.632	0.687	0.688	0.720	0.636	0.631
	Sensitivity	0.689	0.446	0.614	0.643	0.690	0.696	0.726	0.636	0.644
	PPV	0.688	0.448	0.622	0.635	0.698	0.691	0.728	0.656	0.628
	NPV	0.759	0.535	0.702	0.719	0.760	0.744	0.805	0.736	0.700
XGB (XGBoost)	Accuracy	0.674	0.553	0.637	0.727	0.628	0.614	0.712	0.651	0.584
	AUROC	0.655	**0.512**	0.593	0.737	0.718	0.624	0.777	0.732	0.648
	F1-score	0.672	0.556	0.638	0.727	0.622	0.608	0.711	0.644	0.586
	Sensitivity	0.674	0.553	0.637	0.727	0.628	0.614	0.712	0.651	0.584
	PPV	0.676	0.565	0.646	0.734	0.634	0.605	0.720	0.655	0.592
	NPV	0.754	0.638	0.724	0.812	0.679	0.701	0.790	0.688	0.687
LGBM (Light GBM)	Accuracy	0.651	0.432	0.599	0.764	0.644	0.674	0.773	0.667	0.674
	AUROC	0.666	0.443	**0.606**	0.787	0.687	**0.750**	0.864	0.694	0.758
	F1-score	0.653	0.415	0.597	0.762	0.642	0.660	0.772	0.668	0.673
	Sensitivity	0.651	0.432	0.599	0.764	0.644	0.674	0.773	0.667	0.674
	PPV	0.661	0.482	0.606	0.772	0.684	0.660	0.777	0.700	0.674
	NPV	0.741	0.559	0.692	0.840	0.779	0.738	0.835	0.791	0.746
CAT (Cat Boost)	Accuracy	0.674	0.523	0.591	0.750	0.651	0.689	0.787	0.705	0.742
	AUROC	0.694	0.472	0.567	**0.823**	0.738	0.733	**0.866**	**0.778**	**0.765**
	F1-score	0.673	0.522	0.591	0.751	0.653	0.690	0.785	0.707	0.740
	Sensitivity	0.674	0.523	0.591	0.750	0.651	0.689	0.787	0.705	0.742
	PPV	0.677	0.526	0.595	0.762	0.660	0.694	0.788	0.727	0.746
	NPV	0.754	0.610	0.692	0.832	0.727	0.773	0.843	0.807	0.807

SAD, social anxiety disorder; PERS, the post-event rumination scale; BFNE, the brief fear of negative evaluation; ISS, the internalized shame scale; AUROC, area under the receiver operating characteristic; PPV, positive predictive value; NPV, negative predictive value.

^a^The highest AUROC scores for each clinical and psychological scale are highlighted in bold to denote superior model performance.

^b^The combination of physiological and acoustic features.

**Table 5:** The predictive performance of the four machine learning models on the severe group for generalized anxiety (STAI-State, STAI-Trait, and BAI) using the best parameters from grid search or random search combined with stratified cross-validation.

Variable^a^		Physiological Features			Acoustic Features			Multimodal Features^b^		
		STAI-State	STAI-Trait	BAI	STAI-State	STAI-Trait	BAI	STAI-State	STAI-Trait	BAI
RF (Random Forest)	Accuracy	0.585	0.592	0.582	0.590	0.621	0.668	0.644	0.720	0.705
	AUROC	**0.652**	**0.671**	0.514	0.584	0.718	0.734	0.685	0.772	0.786
	F1-score	0.585	0.596	0.575	0.589	0.621	0.666	0.641	0.719	0.695
	Sensitivity	0.585	0.592	0.582	0.590	0.621	0.668	0.644	0.720	0.705
	PPV	0.620	0.615	0.599	0.592	0.631	0.688	0.652	0.730	0.702
	NPV	0.716	0.702	0.672	0.674	0.704	0.741	0.709	0.795	0.763
XGB (XGBoost)	Accuracy	0.555	0.569	0.553	0.629	0.628	0.721	0.630	0.727	0.689
	AUROC	0.623	0.628	**0.549**	0.690	0.674	0.735	0.693	0.744	0.743
	F1-score	0.557	0.568	0.543	0.620	0.631	0.713	0.627	0.726	0.690
	Sensitivity	0.555	0.569	0.553	0.629	0.628	0.721	0.630	0.727	0.689
	PPV	0.592	0.583	0.562	0.657	0.642	0.719	0.644	0.734	0.691
	NPV	0.688	0.670	0.669	0.722	0.725	0.763	0.708	0.798	0.764
LGBM (Light GBM)	Accuracy	0.562	0.561	0.476	0.660	0.673	0.713	0.683	0.766	0.741
	AUROC	0.599	0.625	0.530	**0.719**	0.708	**0.773**	0.732	**0.819**	0.765
	F1-score	0.565	0.556	0.482	0.656	0.668	0.702	0.679	0.766	0.736
	Sensitivity	0.562	0.561	0.476	0.660	0.673	0.713	0.683	0.766	0.741
	PPV	0.571	0.585	0.505	0.667	0.691	0.729	0.696	0.776	0.746
	NPV	0.649	0.676	0.613	0.729	0.736	0.769	0.755	0.827	0.807
CAT (Cat Boost)	Accuracy	0.538	0.615	0.523	0.689	0.704	0.698	0.682	0.750	0.719
	AUROC	0.598	0.624	0.547	0.716	**0.754**	0.770	**0.740**	0.796	**0.809**
	F1-score	0.539	0.615	0.516	0.687	0.701	0.692	0.681	0.751	0.721
	Sensitivity	0.538	0.615	0.523	0.689	0.704	0.698	0.682	0.750	0.719
	PPV	0.562	0.627	0.523	0.689	0.711	0.704	0.696	0.760	0.724
	NPV	0.650	0.719	0.631	0.741	0.765	0.746	0.745	0.817	0.794

STAI-State, the state-trait anxiety inventory-state; STAI-Trait, the state-trait anxiety inventory-trait; BAI, the beck anxiety inventory; AUROC, area under the receiver operating characteristic; PPV, positive predictive value; NPV, negative predictive value.

a The highest AUROC scores for each clinical and psychological scale are highlighted in bold to denote superior model performance.

b The combination of physiological and acoustic features.

In categorizing the core symptoms of SAD, the prediction of CatBoost model for the severe K-SPS group was notable, achieving an AUROC of 0.852. This was closely followed by the prediction of XGBoost model for the severe K-LSAS group with an AUROC of 0.843, and the prediction of CatBoost for the severe groups of K-SADS and K-SIAS with AUROCs of 0.822 and 0.808, respectively. Regarding the cognitive symptoms of SAD, CatBoost predictions for the severe group of PERS, BFNE, and ISS were marked by AUROCs of 0.866, 0.778, and 0.765, respectively. In the context of generalized anxiety, the prediction of LightGBM model for the severe group of STAI-Trait was the most accurate, with an AUROC of 0.819, whereas the predictions of CatBoost for those of BAI and STAI-State were characterized by AUROCs of 0.809 and 0.740, respectively.

**Figure 2:**
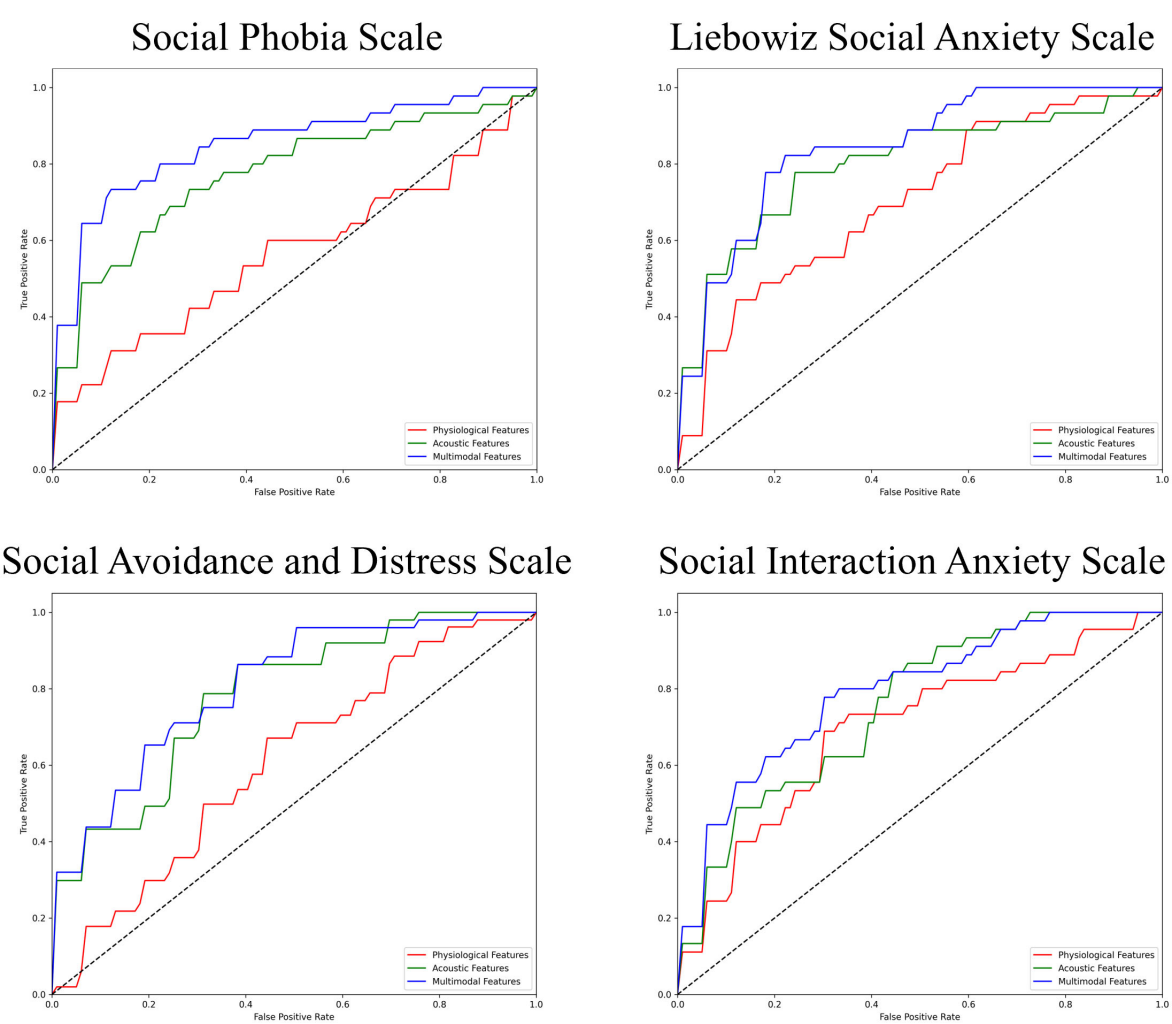
ROC curves of the best prediction on the severe group for core symptoms of SAD. ROC, receiver operating characteristic; SAD, social anxiety disorder. For the Social Phobia Scale, Liebowitz Social Anxiety Scale, Social Avoidance and Distress Scale, and Social Interaction Anxiety Scale, we used the Korean versions.

**Figure 3:**
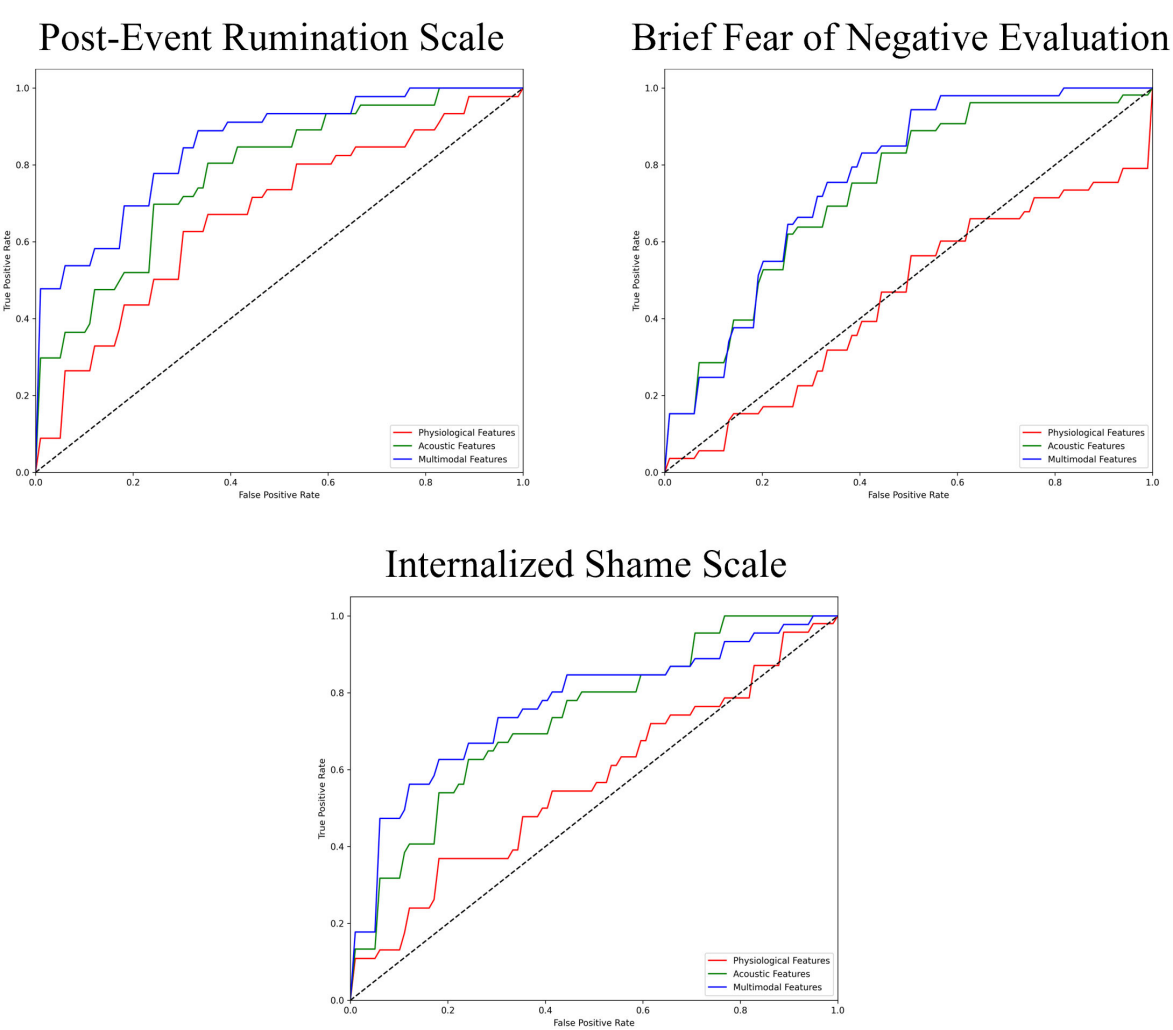
ROC curves of the best prediction on the severe group for cognitive symptoms of SAD. ROC, receiver operating characteristic; SAD, social anxiety disorder.

**Figure 4:**
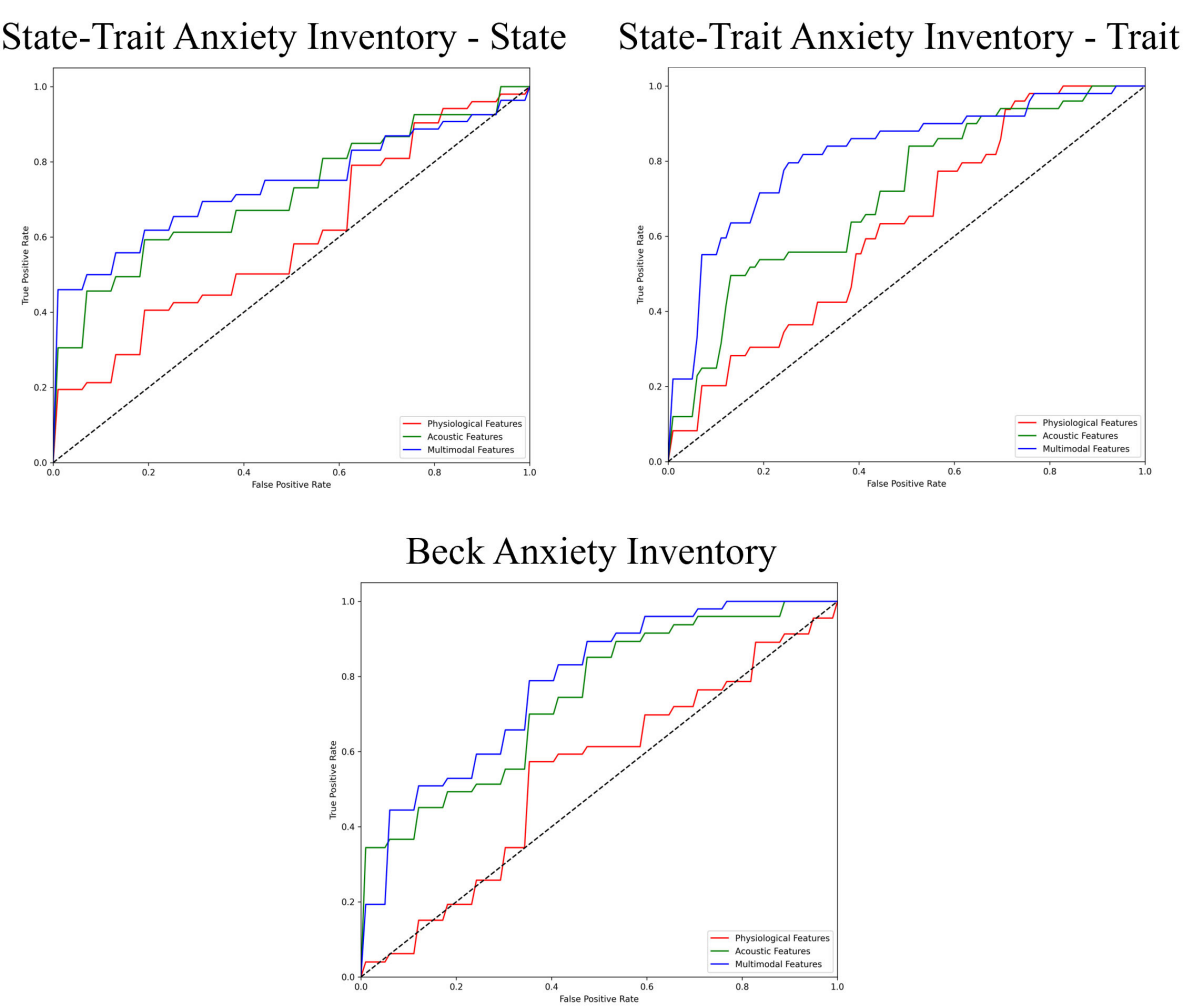
ROC curves of the best prediction on the severe group for generalized anxiety. ROC, receiver operating characteristic.

**Figure 5:**
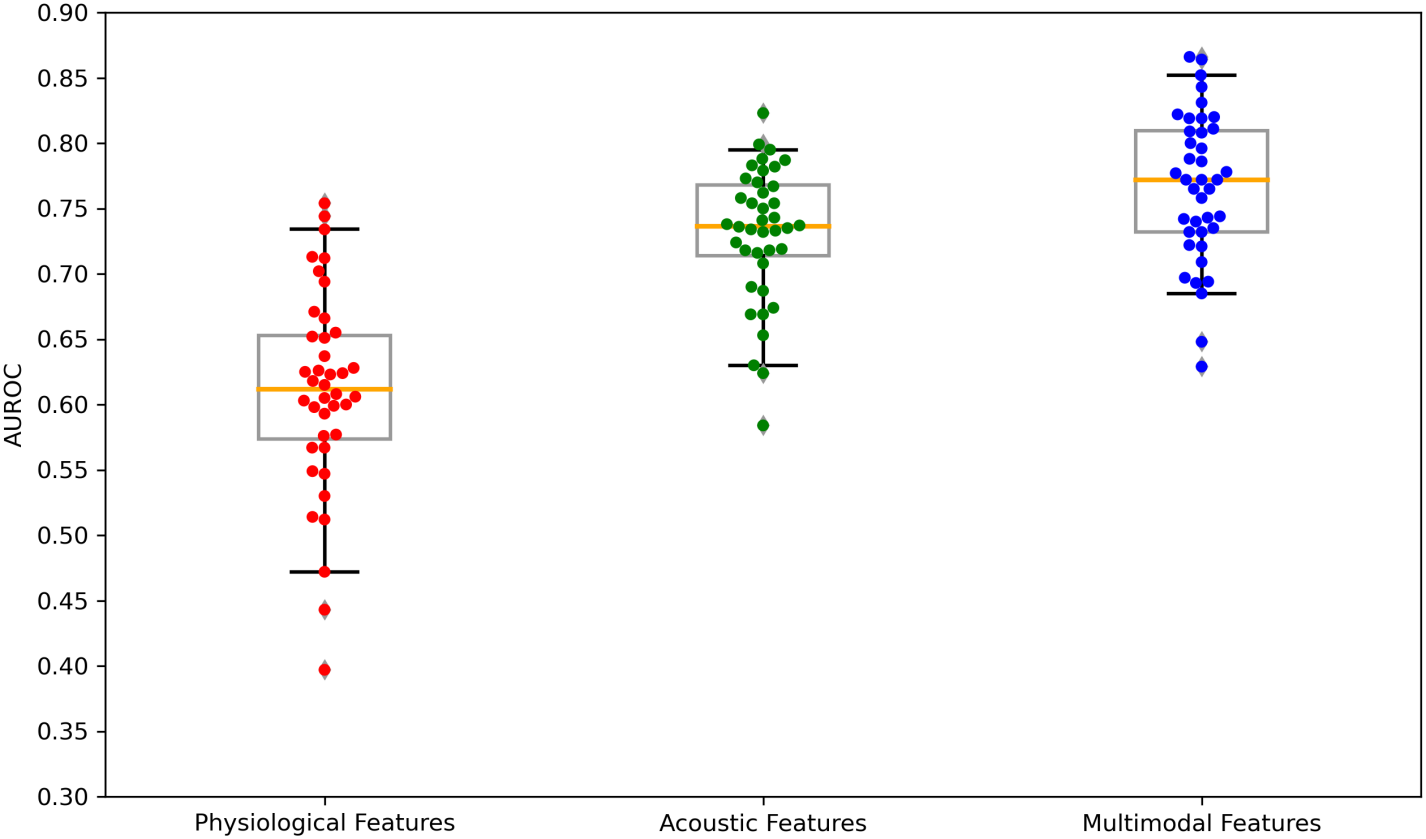
Boxplots of the AUROC scores across feature sets: physiological features, acoustic features, and multimodal features. AUROC, area under the receiver operating characteristics. Each dot is a data point in the performance metric, and the yellow line is the median value.

**Figure 6:**
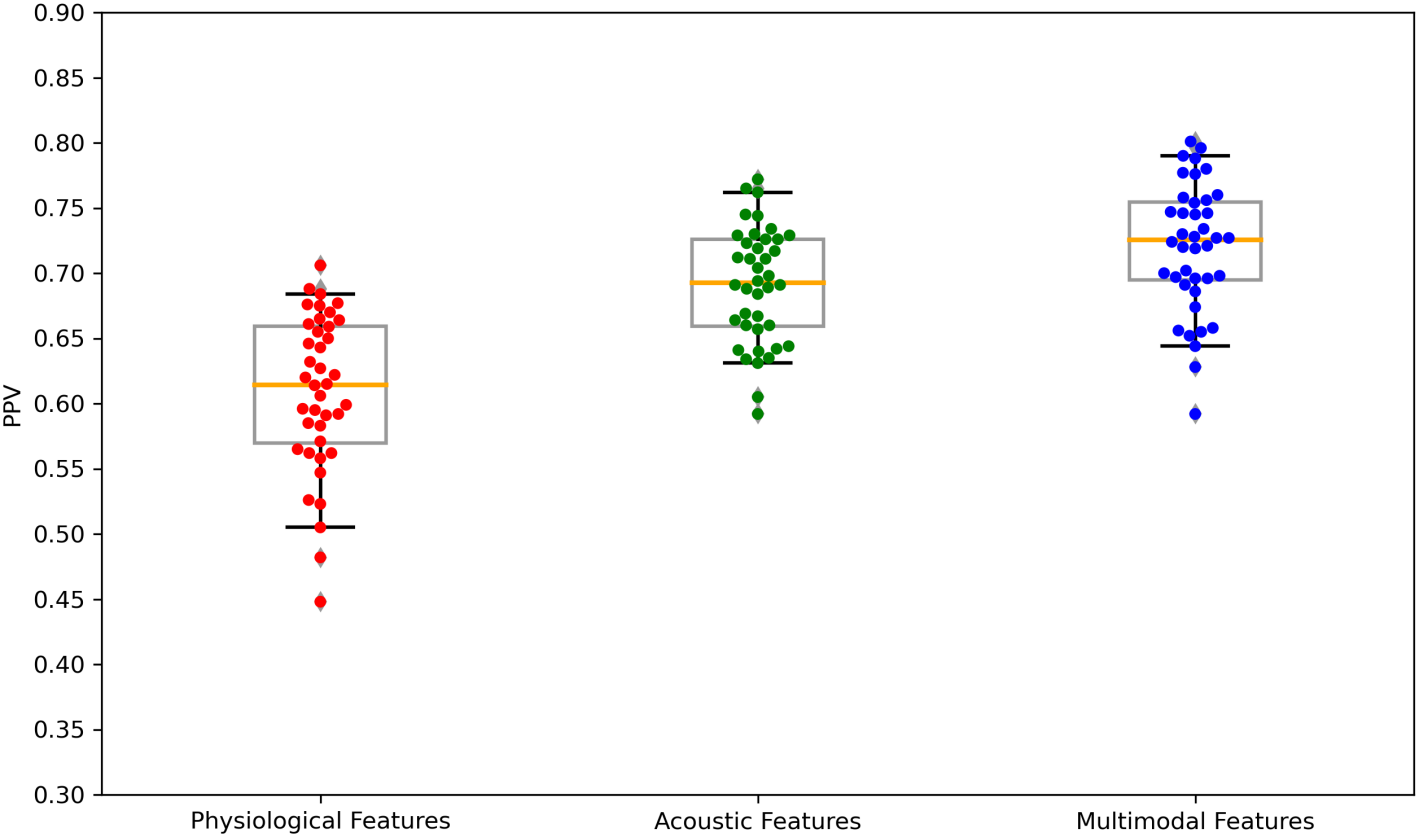
Boxplots of the PPV scores across feature sets: Physiological features, acoustic features, and multimodal features. PPV, positive predictive value. Each dot is a data point in the performance metric, and the yellow line is the median value.

The performance of the top-scoring models, as visualized by receiver operating characteristic curves, was shown in **Figures 2**–**4**. A thorough analysis of the performance metrics across various scales, focusing on the AUROC, revealed a clear pattern: ML models utilizing acoustic features outperformed those based solely on physiological features. This performance gap was further amplified in the models that integrated multimodal features. These results were also evident in the visualizations of AUROC and PPV in **Figures 5**, **6**.

The results of validating the best parameter models using LOOCV were presented in **Table 6**. With AUROC ranging from 0.725 to 0.835, the performance was slightly lower compared to the stratified cross-validation results, but the best prediction performance based on the AUROC was achieved using models that utilized multimodal features, and the same trend was observed in the results of the LOOCV.

**Table 6:** The predictive performance of the four machine learning models on the severe group for all clinical and psychological scales using leave-one-out cross-validation of best parameter models.

Variable^a^	Physiological Features				Acoustic Features				Multimodal Features^b^			
Core Symptoms of SAD	K-SPS	K-LSAS	K-SADS	K-SIAS	K-SPS	K-LSAS	K-SADS	K-SIAS	K-SPS	K-LSAS	K-SADS	K-SIAS
Accuracy	0.629	0.712	0.636	0.667	0.795	0.705	0.659	0.735	0.758	0.727	0.697	0.750
AUROC	0.537	0.728	0.611	0.686	0.801	0.735	0.733	0.720	**0.826**	**0.799**	**0.782**	**0.780**
F1-score	0.592	0.707	0.625	0.648	0.789	0.690	0.638	0.724	0.742	0.724	0.682	0.735
Sensitivity	0.629	0.712	0.636	0.667	0.795	0.705	0.659	0.735	0.758	0.727	0.697	0.750
PPV	0.588	0.704	0.624	0.646	0.791	0.690	0.647	0.725	0.752	0.722	0.690	0.743
NPV	0.676	0.763	0.677	0.713	0.812	0.740	0.680	0.765	0.767	0.780	0.711	0.765

Cognitive Symptoms of SAD	PERS	BFNE	ISS	PERS	BFNE	ISS	PERS	BFNE	ISS
Accuracy	0.727	0.462	0.689	0.758	0.652	0.705	0.758	0.667	0.735
AUROC	0.719	0.410	0.690	0.790	0.668	0.687	**0.825**	**0.725**	**0.780**
F1-score	0.716	0.452	0.672	0.737	0.634	0.684	0.742	0.643	0.720
Sensitivity	0.727	0.462	0.689	0.758	0.652	0.705	0.758	0.667	0.735
PPV	0.717	0.445	0.673	0.760	0.642	0.691	0.754	0.662	0.726
NPV	0.755	0.545	0.723	0.755	0.670	0.728	0.765	0.673	0.752

Generalized Anxiety	STAI-State	STAI-Trait	BAI	STAI-State	STAI-Trait	BAI	STAI-State	STAI-Trait	BAI
Accuracy	0.614	0.606	0.629	0.682	0.720	0.758	0.682	0.765	0.773
AUROC	0.573	0.628	0.572	0.734	0.737	0.833	**0.750**	**0.800**	**0.835**
F1-score	0.598	0.595	0.618	0.674	0.711	0.743	0.670	0.762	0.759
Sensitivity	0.614	0.606	0.629	0.682	0.720	0.758	0.682	0.765	0.773
PPV	0.598	0.591	0.614	0.675	0.712	0.755	0.674	0.762	0.773
NPV	0.653	0.667	0.691	0.711	0.745	0.762	0.702	0.795	0.772

SAD, social anxiety disorder; K-SPS, the Korean version of the social phobia scale; K-LSAS, the Korean version of the liebowitz social anxiety scale; K-SADS, the Korean version of the social avoidance and distress scale; K-SIAS, the Korean version of the social interaction anxiety scale; PERS, the post-event rumination scale; BFNE, the brief fear of negative evaluation; ISS, the internalized shame scale; STAI-State, the state-trait anxiety inventory-state; STAI-Trait, the state-trait anxiety inventory-trait; BAI, the beck anxiety inventory; AUROC, area under the receiver operating characteristic; PPV, positive predictive value; NPV, negative predictive value.

^a^The highest AUROC scores for each clinical and psychological scale are highlighted in bold to denote superior model performance.

^b^The combination of physiological and acoustic features.

### 3.3 Influential factors for predictions using SHAP values

**Figure 7:**
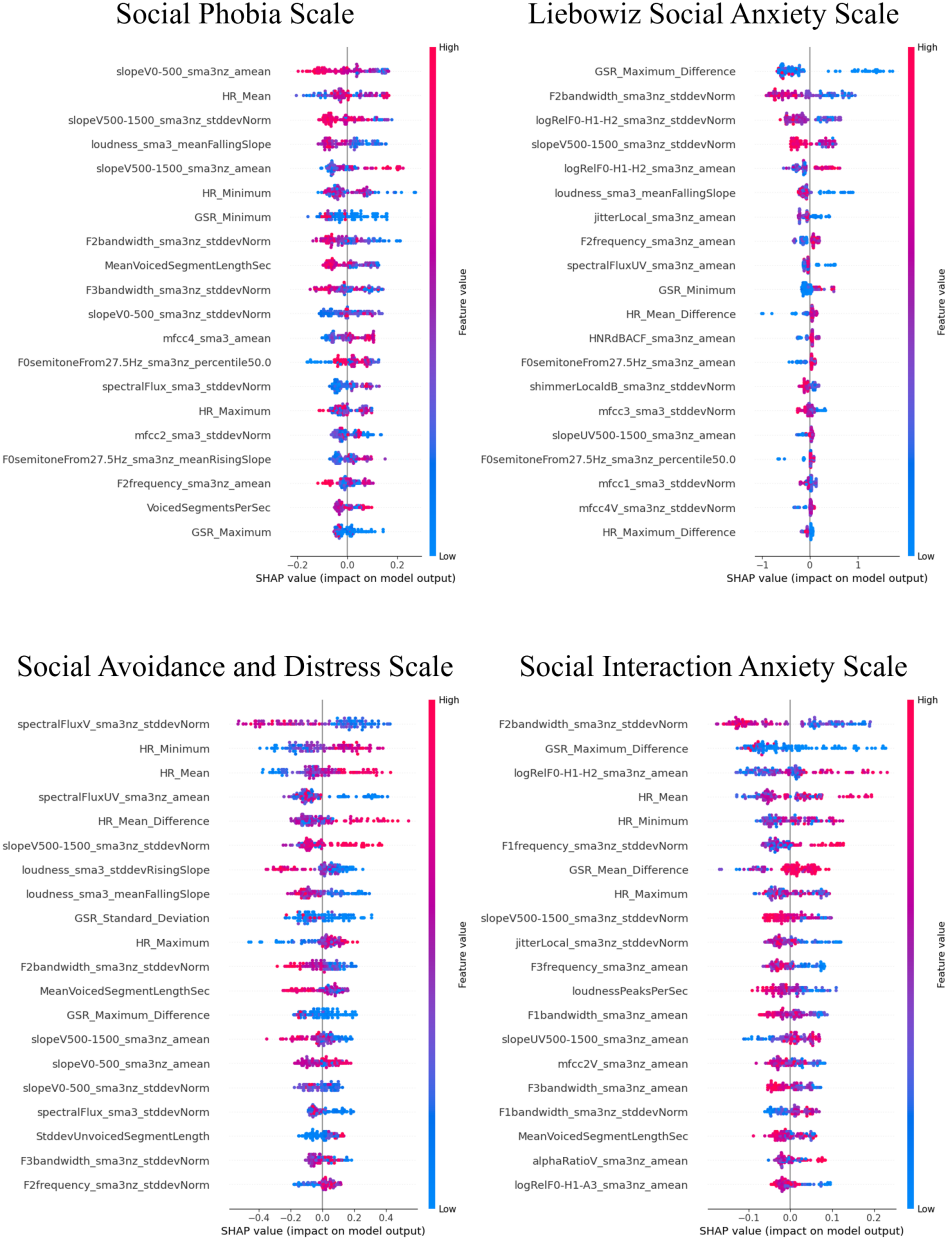
SHAP analysis: multimodal features impact on core symptoms of SAD severity prediction. SHAP, shapley additive explanations; SAD, social anxiety disorder. For the Social Phobia Scale, Liebowitz Social Anxiety Scale, Social Avoidance and Distress Scale, and Social Interaction Anxiety Scale, we used the Korean versions. This visual representation clearly demonstrated the impact of specific characteristics of multimodal features on model predictions across a range of clinical and psychological scales, with features listed in order of importance from the top of the y-axis.

**Figure 8:**
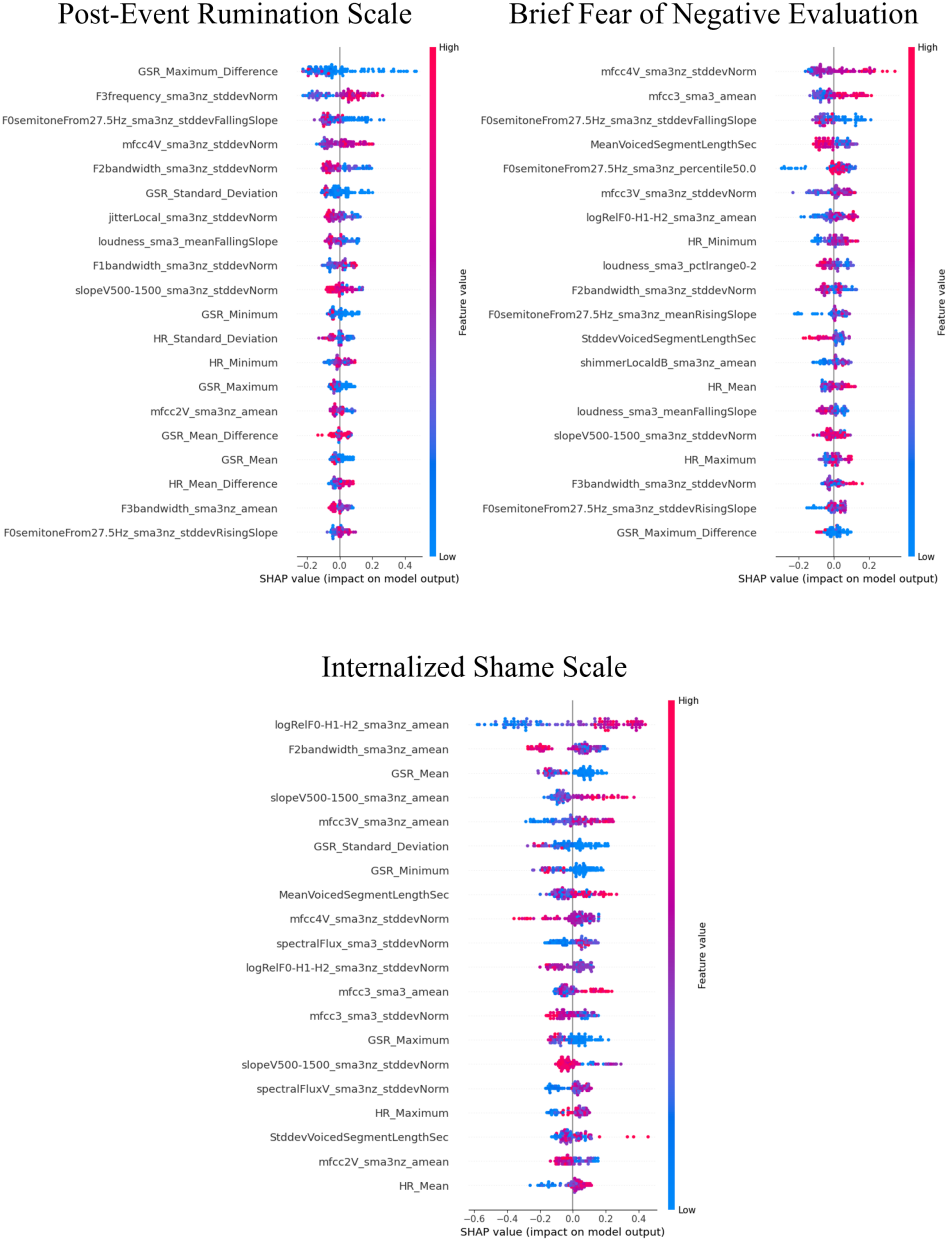
SHAP analysis: multimodal features impact on cognitive symptoms of SAD severity prediction. SHAP, shapley additive explanations; SAD, social anxiety disorder. This visual representation clearly demonstrated the impact of specific characteristics of multimodal features on model predictions across a range of clinical and psychological scales, with features listed in order of importance from the top of the y-axis.

**Figure 9:**
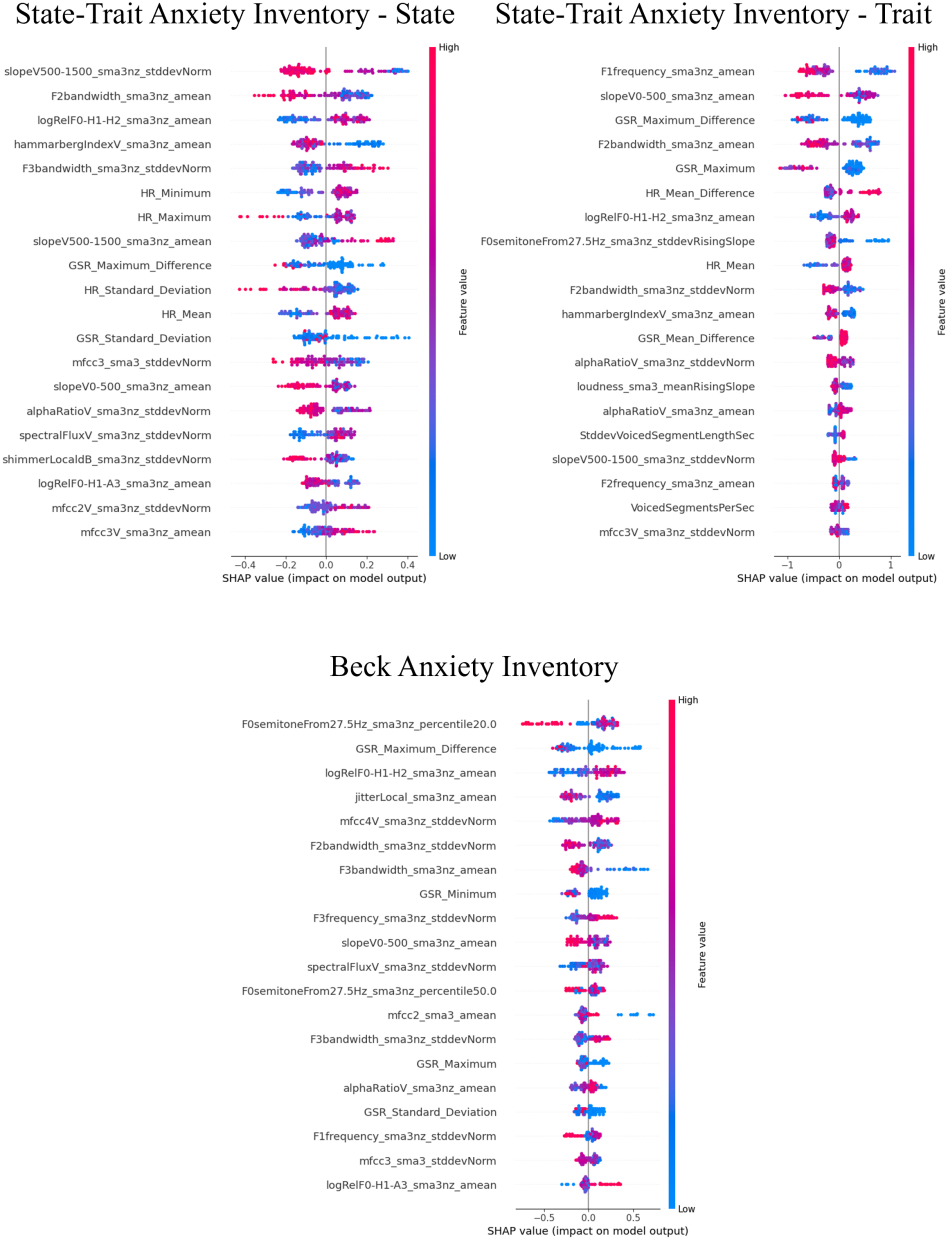
SHAP analysis: multimodal features impact on generalized anxiety severity prediction. SHAP, shapley additive explanations; SAD, social anxiety disorder. This visual representation clearly demonstrated the impact of specific characteristics of multimodal features on model predictions across a range of clinical and psychological scales, with features listed in order of importance from the top of the y-axis.

The SHAP values for the models that demonstrated superior performance with multimodal features are shown in **Figures 7**–**9**. Overall, while acoustic features generally had a greater influence, the Liebowitz Social Anxiety Scale and the Post-Event Rumination Scale showed that GSR had the most significant impact on the model’s predictions.

For the core symptoms of SAD, examining the top five features reveals that, aside from the Liebowitz Social Anxiety Scale, the mean and minimum values of HR exerted a significant influence on the predictions for the other three scales. In contrast, for the cognitive symptoms of SAD and the generalized anxiety, acoustic features played a major role in influencing the model’s predictions, apart from GSR.

## 4 Discussion

This study aimed to examine the clinical utility of ML models using acoustic and physiological data, as well as combined multimodal data from VR sessions, as input data for the prediction of multifaceted SAD symptoms. The focus of this study was to address the potential of using multimodal features to build an ML model. Although models for the real time detection of the mental states of patients with anxiety have been widely developed, they have received relatively little attention in the development of symptom prediction models. This study aimed to identify individuals with severe symptoms in each SAD symptom domain. In general, study findings shed light on ML-driven identification of individuals who may not benefit from specific treatment settings, thereby helping clinicians have insights into ways to develop another approach for the treatment strategy.

In the burgeoning field of digital health, VR applications showcase their ability to elicit and modulate psychological responses in real time and integrate these data within an ML framework. To this end, ML-combined VRET systems have been developed to be predominantly capable of automatically detecting patients’ levels of anxiety (13, 64–66), arousal (12) and stress (67) in real-time, and to change subsequent scenarios depending on the detected patients’ state [i.e., VR-based biofeedback (12, 13)]. Concurrently, to extend this literature, the present study introduces a novel predictive model encompassing a range of SAD symptom facets and reports overall good performance with an average AUROC of 80.6% for multimodal ML models. It presents a diverse array of performance metrics across feature utilizations. This emphasizes the significance of AUROC as a measure of model performance at all threshold levels, providing insights into the influence of features on models that demonstrate high AUROC scores. Building on these findings, the CatBoost model demonstrated notable performance across various symptom domains of SAD, particularly in predicting severe cases of K-SPS and PERS, with AUROCs of 0.852 and 0.866, respectively. This superior performance can be attributed to CatBoost’s advanced algorithmic features, including its use of randomized permutations during training to mitigate overfitting and its capacity to effectively model high-order feature interactions. These characteristics are especially advantageous in multimodal datasets, where complex relationships between diverse features, such as psychological and physiological measures, must be captured (62). Overall, the results offer new promise for the development of ML models for classifying individuals at risk of not responding to ongoing treatment via the detection of those reporting greater severity in each symptom domain over therapy sessions.

The slight performance differences observed between stratified k-fold cross-validation and LOOCV suggest that the choice of validation method can influence model evaluation outcomes. While LOOCV provides a less biased estimate of performance by leveraging all available data for training, it can be computationally demanding. Stratified k-fold, on the other hand, mitigates potential class imbalance in the test folds, making it more suitable for datasets with uneven distributions. These findings underscore the need for methodologically robust approaches when evaluating machine learning models, particularly in small-scale studies like the present one (68). Future research should further explore how validation strategies influence generalizability and interpretability in similar contexts.

From an affective neuroscience perspective, as affective states are accompanied by significant physiological changes in human body, such as brain, heart, skin, blood flow, muscles and organs, their responses have been used as objective markers for identifying current mental states (69). In light of this, studies on VRET for patients with SAD have assessed physiological signals, particularly HR and GSR indices, for assessing anxiety states. Prior studies have shown that HR in patients with SAD significantly changed when confronting a conversation with avatars (70) and delivering a speech with increased virtual audiences (71). In terms of electrodermal activity, increased responses were synchronized with both increased negative affect and decreased positive affect (72) and observed when seeing a face with direct gaze (73). Our finding showing that the model utilizing physiological data alone achieved AUROC up to 0.754 is in alignment with previous findings.

The measurement of mental state has been significantly enhanced by leveraging diverse data streams. For instance, previous studies have presented ML models for detecting real time anxiety in patients by measuring the HR, GSR, blood volume pressure, skin temperature, and electroencephalography (13, 17, 64, 66). However, given that there have been few ML investigations on the potential of combining VRET and multimodality, this study was designed to describe an ML framework combined with multiple sources of information for the identification of at-risk patients. Consequently, the detection performance was superior when acoustic and physiological features were integrated. Specifically, AUROC ranged from 85.2% to 74.0%, comparable to previously reported values [i.e., accuracy, 89.5% (65), 86.3% (66), and 81% (64); AUROC, 0.86% (74)]. Regarding the notably powerful prediction for SPS, it is plausible that our VR content, which provides a self-introduction, could be particular to evaluating scrutiny fear (41), which is assessed by SPS, suggesting that the proposed algorithms might not be accurately predicted in other VRET scenarios. In summary, integrating multimodal data sources can significantly enhance our understanding of the ongoing patient symptomatology trajectories from a holistic perspective.

The results revealed that models utilizing acoustic features showed superior classification performance compared with those utilizing physiological features. Moreover, the interpretation provided by SHAP to obtain an overview of the important features in models with multimodal data highlighted that most predictors across a set of SAD symptoms were derived from audio data. Similarly, a previous study (75) reported that acoustic measures were better predictors of VRET effectiveness for mitigating public speaking anxiety than physiological measures. These findings corroborate an earlier finding that while physiological data (i.e., HR) are only predictive of task-induced stress levels in children with ASD, acoustic data are more predictive of ASD severity in both ASD and typically developing populations (76). Overall, physiological responses represent transient states of intense emotion (e.g., anxiety and stress), whereas voice acoustic changes may be more closely linked to the pathological development of psychiatric disorders.

Supporting this speculation, physiological responses such as HR and GSR are controlled by the autonomic nervous system, which is a part of the peripheral nervous system responsible for regulating involuntary physiological processes (77). Moreover, according to the James–Lange theory (78), emotional experience is largely due to the experience of physiological changes. Therefore, physiological responses strongly predict momentary emotional states. However, speech production involves not only a sound source (i.e., the larynx) coupled to a sound filter represented by the vocal tract airways [i.e., the oral and nasal cavities (79)], but also the engagement of widespread brain regions including several areas of the frontal lobe as well as cortico-subcortical loops traversing the thalamus and basal ganglia (80, 81). In particular, regions such as the amygdala, orbitofrontal cortex, and anterior cingulate cortex are involved in encoding the emotional valence of speech (82, 83). Meanwhile, dysfunction of such areas has been widely reported in patients with SAD (84, 85), suggesting a close link between acoustic characteristics and symptomatology of patients with SAD. In summary, our findings strongly support the integration of voice data to enhance the SAD status prediction.

An alternative explanation of the results regarding the accentuated power of acoustic over physiological data is that providing a speech in public, including a self-introduction, requires the engagement with active efforts to mitigate global physical and physiological changes that occur in the body, such as muscles, heart, and other important organs, in response to social threat and its consequence could be reflected on diverse voice metrics. For example, in terms of fundamental frequency (F0), one of the properties used in this study, its heightened value can be explained by increased vocal cord tension which is a plausible consequence of an increase in overall muscle tone, suggesting that freezing in response to social threat could lead to F0 alteration, alongside with increases in overall muscular tension (86). Similarly, the increase in lung pressure as a part of the body’s fight-flight response, mediated by the central nervous system regulation of the hypothalamic–pituitary–adrenal axis stress response, could also affect the increase in vocal intensity, as well as the delay in voice-onset-time (87, 88). Therefore, utilizing a variety of acoustic indices may provide more information about the pathological aspects of social anxiety than integrating a limited number of physiological indices, such as electrodermal and cardiovascular responses; yet, more studies are needed to understand which types of features are more critical than others for predicting SAD symptom trajectories.

Considering the generalizability of the study, it is important to note that our results were obtained from a relatively small sample of young adults with SAD. While our findings are promising, the limited sample size and specific demographic characteristics of our participants constrain the broad applicability of our models. Further research with larger and more diverse samples, involving patients with heterogeneous symptoms, is necessary to validate the robustness and reliability of these models across different populations with varying symptom profiles. Studies with other age ranges, such as adolescents and middle-aged and older adults with SA needed to improve the degree of generalization of the proposed ML models. Considering our findings from Korean sample comprising people who are well educated with relatively secure socioeconomic status, further external validation is required in order to generalize to other populations with different cultures and races. Moreover, implementing the proposed ML algorithms in other VR scenarios (e.g., providing public speeches or role-playing conversations) could be very challenging due to specificity of VR scenario employed in this study. Considering the scenario was specific to situation of a self-introduction in front of new colleagues, the proposed ML algorithms should be further validated with other anxiety-inducing contexts, such as shopping in a grocery store, conducting a job interview, providing a presentation in a business meeting, and attending a party. It is recognized that the reliance on binary classification limits the depth of analysis, particularly considering the complexity of SAD symptoms. Adopting a multiclass classification approach could provide a more nuanced perspective on symptom severity, thereby improving the capability to track symptom progression and tailor interventions more precisely. Future research should focus on developing and evaluating multiclass models to capture these varying severity levels, which would contribute significantly to precision psychiatry. Lastly, while physiological features such as HR and GSR provide valuable insights, the absence of continuous time-series analysis limits our understanding of dynamic symptom patterns. This limitation could be addressed in future research through the application of temporal data analysis techniques. Additionally, as HR data was not collected at a frequency of at least 100 Hz, performing a heart rate variability (HRV) analysis was not feasible, representing a limitation of the current study. Considering the important role of HRV as a biomarker to measure regularity of HR fluctuations (i.e., HR coherence) and as an indicator of autonomic regulation and the existing literature on associations not only between deep breathing and increased HRV, but also between pathological anxiety and reduced HRV, further incorporating HRV into the model may help improve predictive performance (89–92). Future research should incorporate high-frequency physiological measurements to facilitate HRV analysis and other temporal evaluations. Furthermore, incorporating multifaceted analyses of HR, GSR, and acoustic signals is recommended to develop a more comprehensive understanding of subjects’ responses over time. Moreover, integrating temporal analysis into real-time, adaptive VR therapy bridges the gap between static assessments and dynamic, patient-specific interventions. By leveraging temporal patterns, such as fluctuations in physiological and acoustic features, real-time adaptation of VR scenarios becomes possible.

Having carefully considered the challenges and limitations highlighted above, we present an abstract concept of ML-driven symptom prediction during mental health treatment, thereby helping clinicians follow patients’ therapeutic responses across therapy sessions without requiring a time-consuming evaluation procedure (i.e., traditional pen-and-paper assessment). The proposed concept will allow clinicians to explore whether patients respond to treatment, leading to important insights and providing the first steps toward precision psychiatry.

## Data Availability

The datasets presented in this article are not readily available because of privacy concerns. Requests to access the datasets should be directed to david0203@gmail.com.

## Footnotes

1
https://www.youtube.com/@socialanxietydisordervrtre8109.
