# Supplementary material for: Machine learning prediction of anxiety symptoms in social anxiety disorder: utilizing multimodal data from virtual reality sessions
Source: Front Psychiatry. 2025 Jan 7;15:1504190. doi: 10.3389/fpsyt.2024.1504190 (PMC11784525; doi:10.3389/fpsyt.2024.1504190)
Supplement: Supplementary file 1 [file Table1.docx]

Supplementary Material

# Supplementary Tables: Table S1 ~ S10

**Table S1.** Acoustic Features Classification and Description via eGeMAPS.

| **Parameters** | **Definition** | **Measure** |
| --- | --- | --- |
| Frequency(F0 ~ 3) | The fundamental frequency of the speech signal | Measure the fundamental frequency and its statistical characteristics such as mean, standard deviation, and percentiles. |
| Loudness | The perceived sound intensity | Quantify the loudness level and its variation across the speech signal. |
| Spectral Flux | Changes in the power spectrum of a signal | Measure the rate of change in the spectral energy distribution. |
| MFCCs  (Mel-Frequency Cepstral Coefficients) | Coefficients that collectively make up an MEL-frequency cepstrum | Capture the short-term power spectrum of sound and represent the spectral properties of the speech signal. |
| Jitter | Frequency variation from cycle to cycle | Assess the variation in frequency, reflecting the stability of vocal fold vibration. |
| Shimmer | Amplitude variation from cycle to cycle | Evaluate the variation in amplitude, indicating the stability of vocal fold vibration. |
| HNR  (Harmonics-to-Noise Ratio) | The ratio of harmonic energy to noise energy in a speech signal | Analyze the ratio of harmonic components to noise, reflecting voice quality. |
| Harmonic Differences | The differences in energy between specific harmonics in the voice | Assess the relative energy levels between fundamental and harmonic frequencies. |
| Formants | Resonant frequencies of the vocal tract | Measure the center frequency and bandwidth of vocal tract resonances, particularly focusing on the first three formants. |
| Alpha Ratio | The ratio of energy in two specific frequency bands | Evaluate the spectral balance between high and low frequencies in the voice. |
| Hammarberg Index | The ratio of the highest energy peak in the 0-2 kHz region to the strongest energy peak in the 2-5 kHz region | Measure the relative dominance of high versus low-frequency energy in the voice. |
| Spectral Slope | The decline in spectral energy across frequency bands | Assess the tilt of the spectral energy distribution from low to high frequencies. |
| Loudness Peaks | The rate of occurrence of loudness peaks in speech | Measure the frequency of loudness peaks over time. |
| Voiced/Unvoiced Segments | The segments of speech where vocal folds vibrate (voiced) and do not vibrate (unvoiced) | Quantify the number and duration of voiced and unvoiced segments in the speech. |
| Equivalent Sound Level | The average loudness level of the speech signal | Measure the average energy of the voice over the duration of the recording. |
| **Abbreviation**: eGeMAPS, the extended Geneva minimalistic acoustic parameter set.  ***Note***: eGeMAPS and minimalistic parameter set synopsis: The eGeMAPS builds upon the core 18 low-level descriptors (LLDs) with an additional 26 parameters, including mel-frequency cepstral coefficients (MFCCs 1-4) and spectral flux metrics for detailed vocal trait analysis. Combined, eGeMAPS provides 88 parameters, offering a rich spectrum of dynamic, frequency, and spectral insights for advanced emotion and speech analysis. The fundamental set includes frequency-related measures like pitch, jitter, formants, energy dynamics such as shimmer and loudness, and spectral attributes including alpha ratio and spectral slopes. Functionals like arithmetic mean and coefficient of variation applied to these LLDs yield 62 parameters, with temporal features further enriching the dataset for comprehensive vocal profiling. | | |

**Table S2.** Description of Physiological Features (HR and GSR).

| **Parameter** | **Definition** | **Measure** | **Application (HR & GSR)** |
| --- | --- | --- | --- |
| Mean | The average value of the dataset | Sum of all data values divided by the number of data points | The average for each HR and GSR  level over a period |
| Standard Deviation | The degree of variation of data values from the mean | Square root of the average of the squared deviations from mean | The Variability for each HR and GSR levels |
| Maximum | The largest value in the dataset | The highest data value | The highest value for each HR and GSR level recorded |
| Minimum | The smallest value in the dataset | The lowest data value | The lowest value for each HR and GSR level recorded |
| Maximum Difference | The largest change between consecutive data points | The maximum difference between consecutive data points | The largest change in HR and GSR level between measures |
| Mean Difference | The average change between consecutive data points | The average difference between consecutive data points | The average change in HR and GSR level between measures |
| **Abbreviations**: HR, heart Rate; GSR, galvanic skin response.  ***Note***: A total of 12 physiological features were utilized, comprising six statistical characteristics each for HR and GSR, for machine learning training. | | | |

**Table S3.** Hyperparameter Ranges for Grid Search and Random Search.

| **Search Method** | **Model** | **Hyperparameters** | **Range** |
| --- | --- | --- | --- |
| Grid | Random Forest | n_estimators | 10, 50, 100 |
|  |  | max_depth | None, 10, 20 |
|  | XGBoost | n_estimators | 100, 200 |
|  |  | learning_rate | 0.01, 0.1, 0.2 |
|  | LightGBM | n_estimators | 100, 200 |
|  |  | learning_rate | 0.01, 0.1, 0.2 |
|  | CatBoost | depth | 4, 6, 10 |
|  |  | learning_rate | 0.01, 0.1, 0.2 |
| Random | Random Forest | n_estimators | 10 to 200 (random integer) |
|  |  | max_depth | None, 10, 20, 30 |
|  | XGBoost | n_estimators | 50 to 200 (random integer) |
|  |  | learning_rate | 0.01 to 0.2 (uniform) |
|  | LightGBM | n_estimators | 50 to 200 (random integer) |
|  |  | learning_rate | 0.01 to 0.2 (uniform) |
|  | CatBoost | depth | 4 to 10 (random integer) |
|  |  | learning_rate | 0.01 to 0.2 (uniform) |

**Table S4.** Comparison of Physiological and Acoustic Features between Clustered Groups (higher, middle, and lower).

|  | **Core Symptom of SAD** | | | | | | | | | | |  | **Cognitive Symptom of SAD** | | | | | | | |  | **Generalized Anxiety** | | | | | | | |
| --- | --- | --- | --- | --- | --- | --- | --- | --- | --- | --- | --- | --- | --- | --- | --- | --- | --- | --- | --- | --- | --- | --- | --- | --- | --- | --- | --- | --- | --- |
| **Variables** | **K-SPS** | |  | **K-LSAS** | |  | **K-SAD** | |  | **K-SIAS** | |  | **PERS** | |  | **BFNE** | |  | **ISS** | |  | **STAI_S** | |  | **STAI_T** | |  | **BAI** | |
|  | F or H | *p* |  | F or H | *p* |  | F or H | *p* |  | F or H | *p* |  | F or H | *p* |  | F or H | *p* |  | F or H | *p* |  | F or H | *p* |  | F or H | *p* |  | F or H | *p* |
| **Physiological Features** | | |  |  |  |  |  |  |  |  |  |  |  |  |  |  |  |  |  |  |  |  |  |  |  |  |  |  |  |
| HR_Mean | 11.390 | **<0.001** |  | 3.063 | 0.050 |  | 5.210 | 0.007 |  | 17.880 | **<0.001** |  | 8.921 | 0.012 |  | 2.584 | 0.079 |  | 1.208 | 0.302 |  | 2.620 | 0.077 |  | 9.354 | **0.009** |  | 0.617 | 0.541 |
| HR_Standard_Deviation | 2.933 | 0.231 |  | 2.040 | 0.361 |  | 2.643 | 0.267 |  | 2.291 | 0.318 |  | 2.756 | 0.252 |  | 2.180 | 0.336 |  | 0.074 | 0.964 |  | 5.796 | 0.055 |  | 2.907 | 0.234 |  | 0.531 | 0.767 |
| HR_Maximum | 16.250 | **<0.001** |  | 3.126 | 0.047 |  | 7.941 | 0.019 |  | 16.207 | **<0.001** |  | 8.962 | 0.011 |  | 4.994 | 0.082 |  | 1.248 | 0.536 |  | 3.239 | 0.042 |  | 6.649 | 0.036 |  | 1.395 | 0.498 |
| HR_Minimum | 10.218 | **<0.001** |  | 7.814 | 0.020 |  | 5.052 | 0.008 |  | 8.703 | **<0.001** |  | 6.151 | 0.003 |  | 2.284 | 0.106 |  | 1.012 | 0.366 |  | 2.936 | 0.057 |  | 5.838 | **0.004** |  | 0.444 | 0.642 |
| HR_Maximum_Difference | 2.321 | 0.313 |  | 2.361 | 0.307 |  | 3.889 | 0.143 |  | 9.346 | 0.009 |  | 1.951 | 0.377 |  | 1.104 | 0.576 |  | 0.498 | 0.780 |  | 4.839 | 0.089 |  | 1.794 | 0.408 |  | 2.129 | 0.345 |
| HR_Mean_Difference | 0.016 | 0.992 |  | 1.772 | 0.412 |  | 1.265 | 0.531 |  | 0.710 | 0.701 |  | 2.826 | 0.243 |  | 0.090 | 0.956 |  | 0.959 | 0.619 |  | 1.691 | 0.429 |  | 1.032 | 0.597 |  | 0.184 | 0.912 |
| GSR_Mean | 9.585 | **0.008** |  | 1.664 | 0.435 |  | 11.449 | 0.003 |  | 2.946 | 0.229 |  | 6.336 | 0.042 |  | 1.261 | 0.532 |  | 5.414 | 0.067 |  | 0.766 | 0.682 |  | 7.508 | 0.023 |  | 4.520 | 0.104 |
| GSR_Standard_Deviation | 4.450 | 0.108 |  | 4.383 | 0.112 |  | 17.628 | 0.000 |  | 4.872 | 0.087 |  | 7.696 | 0.021 |  | 0.166 | 0.920 |  | 6.137 | 0.046 |  | 3.558 | 0.169 |  | 8.587 | **0.014** |  | 1.578 | 0.454 |
| GSR_Maximum | 8.865 | **0.012** |  | 1.824 | 0.402 |  | 12.655 | 0.002 |  | 2.851 | 0.240 |  | 6.324 | 0.042 |  | 1.212 | 0.545 |  | 5.614 | 0.060 |  | 0.953 | 0.621 |  | 8.870 | **0.012** |  | 4.745 | 0.093 |
| GSR_Minimum | 10.472 | **0.005** |  | 1.468 | 0.480 |  | 9.872 | 0.007 |  | 2.972 | 0.226 |  | 6.220 | 0.045 |  | 0.949 | 0.622 |  | 4.554 | 0.103 |  | 1.400 | 0.497 |  | 7.147 | 0.028 |  | 4.718 | 0.095 |
| GSR_Maximum_Difference | 6.927 | 0.031 |  | 15.140 | 0.001 |  | 5.574 | 0.062 |  | 12.763 | **0.002** |  | 17.757 | 0.000 |  | 2.803 | 0.246 |  | 3.931 | 0.140 |  | 5.095 | 0.078 |  | 16.440 | **0.000** |  | 7.406 | 0.025 |
| GSR_Mean_Difference | 2.396 | 0.302 |  | 1.836 | 0.399 |  | 1.943 | 0.378 |  | 1.967 | 0.374 |  | 0.429 | 0.807 |  | 1.376 | 0.503 |  | 4.434 | 0.109 |  | 1.280 | 0.527 |  | 3.492 | 0.174 |  | 0.353 | 0.838 |
| **Acoustic Features** | |  |  |  |  |  |  |  |  |  |  |  |  |  |  |  |  |  |  |  |  |  |  |  |  |  |  |  |  |
| F0semitoneFrom27.5Hz_sma3nz_amean | 10.452 | **0.005** |  | 2.459 | 0.292 |  | 3.245 | 0.197 |  | 2.838 | 0.242 |  | 3.371 | 0.185 |  | 0.177 | 0.915 |  | 1.158 | 0.560 |  | 2.468 | 0.291 |  | 17.392 | **<0.001** |  | 0.018 | 0.991 |
| F0semitoneFrom27.5Hz_sma3nz_stddevNorm | 12.110 | **0.002** |  | 2.678 | 0.262 |  | 0.677 | 0.713 |  | 4.176 | 0.124 |  | 2.610 | 0.271 |  | 1.186 | 0.553 |  | 1.140 | 0.566 |  | 0.927 | 0.398 |  | 21.749 | **<0.001** |  | 0.465 | 0.793 |
| F0semitoneFrom27.5Hz_sma3nz_percentile20.0 | 10.375 | **0.006** |  | 3.236 | 0.198 |  | 1.553 | 0.460 |  | 3.443 | 0.179 |  | 2.819 | 0.244 |  | 0.255 | 0.880 |  | 0.383 | 0.826 |  | 1.792 | 0.408 |  | 19.575 | **<0.001** |  | 0.285 | 0.867 |
| F0semitoneFrom27.5Hz_sma3nz_percentile50.0 | 14.924 | **0.001** |  | 1.451 | 0.484 |  | 2.273 | 0.321 |  | 3.170 | 0.205 |  | 3.440 | 0.179 |  | 0.776 | 0.678 |  | 1.101 | 0.577 |  | 2.796 | 0.247 |  | 22.643 | **<0.001** |  | 0.278 | 0.870 |
| F0semitoneFrom27.5Hz_sma3nz_percentile80.0 | 5.516 | 0.063 |  | 0.240 | 0.887 |  | 2.516 | 0.284 |  | 1.025 | 0.599 |  | 3.606 | 0.165 |  | 0.177 | 0.915 |  | 0.880 | 0.644 |  | 2.400 | 0.301 |  | 10.851 | **0.004** |  | 0.346 | 0.841 |
| F0semitoneFrom27.5Hz_sma3nz_pctlrange0-2 | 1.044 | 0.593 |  | 2.371 | 0.306 |  | 0.715 | 0.699 |  | 7.624 | 0.022 |  | 1.642 | 0.440 |  | 0.392 | 0.822 |  | 0.447 | 0.800 |  | 0.216 | 0.897 |  | 6.721 | 0.035 |  | 0.390 | 0.823 |
| F0semitoneFrom27.5Hz_sma3nz_meanRisingSlope | 12.057 | **0.002** |  | 1.067 | 0.586 |  | 0.769 | 0.681 |  | 3.107 | 0.212 |  | 1.434 | 0.488 |  | 6.796 | 0.033 |  | 0.693 | 0.707 |  | 1.586 | 0.452 |  | 6.134 | 0.047 |  | 0.297 | 0.862 |
| F0semitoneFrom27.5Hz_sma3nz_stddevRisingSlope | 10.021 | **0.007** |  | 1.133 | 0.568 |  | 1.388 | 0.500 |  | 4.230 | 0.121 |  | 1.233 | 0.540 |  | 4.261 | 0.119 |  | 0.022 | 0.989 |  | 1.463 | 0.235 |  | 4.088 | **0.019** |  | 1.276 | 0.283 |
| F0semitoneFrom27.5Hz_sma3nz_meanFallingSlope | 5.336 | 0.069 |  | 1.545 | 0.462 |  | 2.086 | 0.352 |  | 3.506 | 0.173 |  | 11.087 | 0.004 |  | 1.799 | 0.407 |  | 1.436 | 0.242 |  | 0.678 | 0.712 |  | 11.434 | **0.003** |  | 0.886 | 0.642 |
| F0semitoneFrom27.5Hz_sma3nz_stddevFallingSlope | 5.574 | 0.062 |  | 2.663 | 0.264 |  | 1.133 | 0.568 |  | 3.373 | 0.185 |  | 11.308 | 0.004 |  | 3.641 | 0.162 |  | 3.628 | 0.163 |  | 0.795 | 0.672 |  | 8.970 | **0.011** |  | 1.943 | 0.378 |
| loudness_sma3_amean | 7.217 | 0.027 |  | 3.023 | 0.221 |  | 2.469 | 0.291 |  | 2.141 | 0.343 |  | 8.454 | 0.015 |  | 7.198 | 0.027 |  | 4.130 | 0.127 |  | 0.605 | 0.739 |  | 2.723 | 0.256 |  | 0.313 | 0.855 |
| loudness_sma3_stddevNorm | 6.038 | 0.049 |  | 3.540 | 0.170 |  | 9.437 | 0.009 |  | 1.335 | 0.513 |  | 3.651 | 0.161 |  | 3.505 | 0.173 |  | 4.877 | 0.087 |  | 3.776 | 0.151 |  | 18.162 | **<0.001** |  | 0.375 | 0.829 |
| loudness_sma3_percentile20.0 | 1.115 | 0.573 |  | 1.306 | 0.520 |  | 7.184 | 0.028 |  | 0.901 | 0.637 |  | 0.139 | 0.933 |  | 3.478 | 0.176 |  | 3.694 | 0.158 |  | 3.009 | 0.222 |  | 3.870 | 0.144 |  | 1.615 | 0.446 |
| loudness_sma3_percentile50.0 | 5.577 | 0.062 |  | 3.965 | 0.138 |  | 6.967 | 0.031 |  | 1.312 | 0.519 |  | 4.811 | 0.090 |  | 6.629 | 0.036 |  | 4.299 | 0.117 |  | 0.393 | 0.822 |  | 7.816 | **0.020** |  | 0.482 | 0.786 |
| loudness_sma3_percentile80.0 | 3.910 | 0.022 |  | 1.342 | 0.265 |  | 0.891 | 0.413 |  | 1.445 | 0.240 |  | 4.791 | 0.010 |  | 3.795 | 0.025 |  | 2.451 | 0.090 |  | 1.295 | 0.278 |  | 1.433 | 0.242 |  | 0.266 | 0.766 |
| loudness_sma3_pctlrange0-2 | 4.519 | **0.013** |  | 1.533 | 0.220 |  | 0.826 | 0.440 |  | 1.707 | 0.185 |  | 5.289 | 0.006 |  | 3.740 | 0.026 |  | 2.605 | 0.078 |  | 1.833 | 0.164 |  | 1.550 | 0.216 |  | 0.488 | 0.615 |
| loudness_sma3_meanRisingSlope | 4.279 | 0.118 |  | 0.673 | 0.512 |  | 6.001 | 0.050 |  | 0.166 | 0.847 |  | 5.163 | 0.076 |  | 3.590 | 0.166 |  | 1.976 | 0.143 |  | 6.944 | 0.031 |  | 2.888 | 0.236 |  | 0.544 | 0.582 |
| loudness_sma3_stddevRisingSlope | 3.400 | 0.183 |  | 0.860 | 0.650 |  | 10.226 | 0.006 |  | 1.332 | 0.514 |  | 3.693 | 0.158 |  | 2.159 | 0.340 |  | 2.499 | 0.287 |  | 7.083 | 0.029 |  | 4.376 | 0.112 |  | 0.545 | 0.761 |
| loudness_sma3_meanFallingSlope | 8.093 | **0.017** |  | 2.519 | 0.284 |  | 7.449 | 0.024 |  | 0.421 | 0.658 |  | 10.609 | 0.005 |  | 4.959 | 0.084 |  | 2.312 | 0.103 |  | 6.260 | 0.044 |  | 1.836 | 0.164 |  | 1.002 | 0.370 |
| loudness_sma3_stddevFallingSlope | 1.702 | 0.186 |  | 0.889 | 0.641 |  | 8.905 | 0.012 |  | 1.210 | 0.546 |  | 5.539 | 0.063 |  | 2.916 | 0.233 |  | 1.489 | 0.229 |  | 2.978 | 0.054 |  | 5.615 | 0.060 |  | 1.001 | 0.606 |
| spectralFlux_sma3_amean | 5.502 | 0.064 |  | 2.414 | 0.299 |  | 3.466 | 0.177 |  | 2.286 | 0.319 |  | 7.553 | 0.023 |  | 5.838 | 0.054 |  | 4.804 | 0.091 |  | 1.135 | 0.567 |  | 2.058 | 0.357 |  | 0.348 | 0.840 |
| spectralFlux_sma3_stddevNorm | 10.517 | **0.005** |  | 1.900 | 0.387 |  | 0.931 | 0.628 |  | 2.569 | 0.277 |  | 2.047 | 0.359 |  | 0.971 | 0.615 |  | 6.232 | 0.044 |  | 3.016 | 0.221 |  | 14.746 | **0.001** |  | 2.118 | 0.347 |
| mfcc1_sma3_amean | 5.942 | 0.051 |  | 3.174 | 0.204 |  | 0.869 | 0.422 |  | 6.117 | **0.003** |  | 1.682 | 0.431 |  | 1.960 | 0.145 |  | 0.047 | 0.977 |  | 1.923 | 0.150 |  | 3.660 | 0.160 |  | 0.518 | 0.772 |
| mfcc1_sma3_stddevNorm | 2.874 | 0.238 |  | 2.295 | 0.317 |  | 3.541 | 0.170 |  | 1.952 | 0.377 |  | 2.779 | 0.249 |  | 5.019 | 0.081 |  | 0.244 | 0.885 |  | 2.474 | 0.290 |  | 5.605 | 0.061 |  | 0.542 | 0.763 |
| mfcc2_sma3_amean | 1.191 | 0.307 |  | 0.881 | 0.417 |  | 0.217 | 0.805 |  | 5.226 | 0.007 |  | 1.191 | 0.307 |  | 2.896 | 0.235 |  | 0.560 | 0.572 |  | 1.534 | 0.464 |  | 1.931 | 0.149 |  | 0.461 | 0.632 |
| mfcc2_sma3_stddevNorm | 1.869 | 0.393 |  | 1.639 | 0.441 |  | 2.067 | 0.356 |  | 2.984 | 0.225 |  | 1.168 | 0.558 |  | 3.437 | 0.179 |  | 0.036 | 0.982 |  | 0.273 | 0.873 |  | 2.872 | 0.238 |  | 0.770 | 0.681 |
| mfcc3_sma3_amean | 4.011 | **0.020** |  | 2.281 | 0.106 |  | 4.051 | 0.020 |  | 1.655 | 0.195 |  | 1.262 | 0.287 |  | 4.554 | 0.012 |  | 6.693 | 0.035 |  | 0.784 | 0.459 |  | 2.804 | 0.064 |  | 0.022 | 0.978 |
| mfcc3_sma3_stddevNorm | 3.335 | 0.189 |  | 5.074 | 0.079 |  | 3.970 | 0.137 |  | 3.464 | 0.177 |  | 0.081 | 0.960 |  | 4.041 | 0.133 |  | 1.731 | 0.421 |  | 4.476 | 0.107 |  | 2.644 | 0.267 |  | 5.118 | 0.077 |
| mfcc4_sma3_amean | 6.833 | **0.002** |  | 2.554 | 0.082 |  | 2.600 | 0.078 |  | 3.052 | 0.051 |  | 1.562 | 0.214 |  | 2.116 | 0.125 |  | 2.681 | 0.072 |  | 0.299 | 0.742 |  | 6.695 | **0.002** |  | 0.235 | 0.791 |
| mfcc4_sma3_stddevNorm | 5.764 | 0.056 |  | 1.339 | 0.512 |  | 4.998 | 0.082 |  | 0.770 | 0.681 |  | 1.428 | 0.490 |  | 2.579 | 0.275 |  | 3.571 | 0.168 |  | 0.589 | 0.745 |  | 5.570 | 0.062 |  | 0.681 | 0.711 |
| jitterLocal_sma3nz_amean | 5.914 | 0.052 |  | 4.749 | 0.093 |  | 1.966 | 0.374 |  | 6.713 | 0.035 |  | 0.876 | 0.645 |  | 0.106 | 0.948 |  | 1.001 | 0.606 |  | 1.065 | 0.587 |  | 14.047 | **0.001** |  | 0.361 | 0.835 |
| jitterLocal_sma3nz_stddevNorm | 5.576 | **0.005** |  | 4.312 | 0.116 |  | 0.446 | 0.800 |  | 6.575 | 0.037 |  | 9.939 | 0.007 |  | 3.017 | 0.221 |  | 1.102 | 0.335 |  | 2.267 | 0.108 |  | 4.096 | **0.019** |  | 0.191 | 0.826 |
| shimmerLocaldB_sma3nz_amean | 12.459 | **0.002** |  | 4.030 | 0.133 |  | 2.623 | 0.269 |  | 3.693 | 0.158 |  | 1.709 | 0.426 |  | 2.340 | 0.310 |  | 0.770 | 0.680 |  | 0.984 | 0.611 |  | 17.412 | **<0.001** |  | 0.071 | 0.965 |
| shimmerLocaldB_sma3nz_stddevNorm | 7.706 | **0.001** |  | 1.898 | 0.154 |  | 0.775 | 0.463 |  | 4.065 | 0.019 |  | 3.449 | 0.035 |  | 1.064 | 0.348 |  | 0.736 | 0.481 |  | 2.023 | 0.136 |  | 7.328 | **0.001** |  | 1.923 | 0.382 |
| HNRdBACF_sma3nz_amean | 8.895 | **0.012** |  | 2.987 | 0.225 |  | 2.110 | 0.125 |  | 3.001 | 0.053 |  | 2.247 | 0.325 |  | 0.073 | 0.964 |  | 0.159 | 0.853 |  | 0.359 | 0.836 |  | 16.486 | **<0.001** |  | 0.043 | 0.979 |
| HNRdBACF_sma3nz_stddevNorm | 6.927 | 0.031 |  | 3.204 | 0.201 |  | 1.036 | 0.596 |  | 2.939 | 0.230 |  | 2.272 | 0.321 |  | 0.902 | 0.637 |  | 3.428 | 0.180 |  | 1.325 | 0.515 |  | 15.186 | **0.001** |  | 0.440 | 0.803 |
| logRelF0-H1-H2_sma3nz_amean | 3.095 | 0.049 |  | 3.062 | 0.050 |  | 1.272 | 0.284 |  | 3.453 | 0.035 |  | 0.588 | 0.557 |  | 3.034 | 0.052 |  | 9.309 | 0.010 |  | 3.003 | 0.053 |  | 8.735 | **<0.001** |  | 2.577 | 0.080 |
| logRelF0-H1-H2_sma3nz_stddevNorm | 0.465 | 0.792 |  | 4.894 | 0.087 |  | 0.723 | 0.697 |  | 1.259 | 0.533 |  | 4.995 | 0.082 |  | 2.260 | 0.323 |  | 3.628 | 0.163 |  | 3.675 | 0.159 |  | 1.158 | 0.560 |  | 0.839 | 0.658 |
| logRelF0-H1-A3_sma3nz_amean | 2.697 | 0.071 |  | 0.291 | 0.748 |  | 0.373 | 0.690 |  | 7.254 | **0.001** |  | 1.433 | 0.489 |  | 2.384 | 0.096 |  | 0.699 | 0.705 |  | 4.983 | 0.008 |  | 4.972 | **0.008** |  | 0.297 | 0.862 |
| logRelF0-H1-A3_sma3nz_stddevNorm | 5.151 | 0.076 |  | 2.800 | 0.247 |  | 2.255 | 0.324 |  | 2.998 | 0.223 |  | 1.700 | 0.427 |  | 0.562 | 0.755 |  | 0.837 | 0.658 |  | 6.200 | 0.045 |  | 15.183 | **0.001** |  | 0.369 | 0.831 |
| F1frequency_sma3nz_amean | 6.696 | **0.002** |  | 0.125 | 0.882 |  | 2.267 | 0.322 |  | 3.845 | 0.146 |  | 4.150 | 0.018 |  | 4.460 | 0.108 |  | 1.679 | 0.191 |  | 0.235 | 0.889 |  | 12.055 | **0.002** |  | 0.851 | 0.653 |
| F1frequency_sma3nz_stddevNorm | 8.488 | **0.014** |  | 7.773 | 0.021 |  | 0.252 | 0.881 |  | 11.626 | **0.003** |  | 8.277 | 0.016 |  | 2.769 | 0.066 |  | 0.100 | 0.951 |  | 2.273 | 0.321 |  | 11.348 | **0.003** |  | 0.942 | 0.625 |
| F1bandwidth_sma3nz_amean | 8.920 | **0.012** |  | 3.809 | 0.149 |  | 0.647 | 0.724 |  | 7.624 | 0.022 |  | 4.868 | 0.088 |  | 6.788 | 0.034 |  | 2.230 | 0.328 |  | 16.015 | 0.000 |  | 14.081 | **0.001** |  | 0.931 | 0.628 |
| F1bandwidth_sma3nz_stddevNorm | 1.064 | 0.587 |  | 2.771 | 0.250 |  | 1.269 | 0.530 |  | 11.058 | **0.004** |  | 6.647 | 0.036 |  | 1.959 | 0.376 |  | 1.308 | 0.520 |  | 2.535 | 0.281 |  | 5.796 | 0.055 |  | 2.219 | 0.330 |
| F1amplitudeLogRelF0_sma3nz_amean | 14.463 | **0.001** |  | 4.757 | 0.093 |  | 2.091 | 0.351 |  | 8.019 | 0.018 |  | 6.354 | 0.042 |  | 4.394 | 0.111 |  | 2.581 | 0.275 |  | 0.244 | 0.885 |  | 7.469 | 0.024 |  | 0.739 | 0.691 |
| F1amplitudeLogRelF0_sma3nz_stddevNorm | 15.217 | **<0.001** |  | 4.139 | 0.126 |  | 1.465 | 0.481 |  | 7.839 | 0.020 |  | 6.402 | 0.041 |  | 5.101 | 0.078 |  | 2.782 | 0.249 |  | 0.606 | 0.739 |  | 5.542 | 0.063 |  | 1.190 | 0.552 |
| F2frequency_sma3nz_amean | 9.335 | **0.009** |  | 0.089 | 0.956 |  | 1.405 | 0.249 |  | 5.089 | 0.079 |  | 7.760 | 0.021 |  | 4.004 | 0.135 |  | 2.089 | 0.128 |  | 1.254 | 0.289 |  | 8.867 | **<0.001** |  | 0.812 | 0.666 |
| F2frequency_sma3nz_stddevNorm | 1.560 | 0.214 |  | 0.850 | 0.430 |  | 3.307 | 0.191 |  | 3.924 | 0.022 |  | 3.569 | 0.031 |  | 1.851 | 0.161 |  | 2.317 | 0.103 |  | 0.214 | 0.807 |  | 0.526 | 0.592 |  | 3.237 | 0.043 |
| F2bandwidth_sma3nz_amean | 3.060 | 0.217 |  | 0.229 | 0.795 |  | 0.032 | 0.968 |  | 0.161 | 0.851 |  | 1.028 | 0.361 |  | 0.995 | 0.608 |  | 2.765 | 0.067 |  | 10.988 | 0.004 |  | 14.086 | **0.001** |  | 1.052 | 0.352 |
| F2bandwidth_sma3nz_stddevNorm | 4.123 | **0.018** |  | 5.369 | 0.006 |  | 2.266 | 0.108 |  | 5.825 | **0.004** |  | 4.434 | 0.014 |  | 1.333 | 0.267 |  | 0.408 | 0.816 |  | 0.011 | 0.989 |  | 7.948 | **0.001** |  | 0.595 | 0.553 |
| F2amplitudeLogRelF0_sma3nz_amean | 13.999 | **0.001** |  | 4.255 | 0.119 |  | 2.280 | 0.320 |  | 6.307 | 0.043 |  | 5.337 | 0.069 |  | 4.240 | 0.120 |  | 2.067 | 0.356 |  | 0.092 | 0.955 |  | 7.390 | 0.025 |  | 0.396 | 0.820 |
| F2amplitudeLogRelF0_sma3nz_stddevNorm | 12.651 | **0.002** |  | 3.124 | 0.210 |  | 1.851 | 0.396 |  | 5.402 | 0.067 |  | 6.248 | 0.044 |  | 5.594 | 0.061 |  | 1.897 | 0.387 |  | 0.407 | 0.816 |  | 4.362 | 0.113 |  | 0.530 | 0.767 |
| F3frequency_sma3nz_amean | 4.171 | **0.018** |  | 0.251 | 0.779 |  | 3.650 | 0.029 |  | 8.602 | 0.014 |  | 2.280 | 0.106 |  | 1.030 | 0.360 |  | 1.568 | 0.212 |  | 0.978 | 0.379 |  | 15.047 | **0.001** |  | 0.555 | 0.758 |
| F3frequency_sma3nz_stddevNorm | 1.305 | 0.521 |  | 1.417 | 0.492 |  | 3.251 | 0.042 |  | 2.284 | 0.106 |  | 10.117 | 0.006 |  | 0.601 | 0.550 |  | 0.308 | 0.857 |  | 5.507 | 0.064 |  | 2.249 | 0.325 |  | 5.627 | 0.060 |
| F3bandwidth_sma3nz_amean | 5.321 | **0.006** |  | 1.144 | 0.322 |  | 0.858 | 0.426 |  | 3.844 | 0.024 |  | 2.605 | 0.272 |  | 3.136 | 0.047 |  | 1.117 | 0.330 |  | 2.912 | 0.058 |  | 2.796 | 0.065 |  | 4.370 | 0.112 |
| F3bandwidth_sma3nz_stddevNorm | 10.561 | **0.005** |  | 0.007 | 0.997 |  | 1.250 | 0.290 |  | 0.591 | 0.744 |  | 0.671 | 0.715 |  | 1.888 | 0.389 |  | 0.502 | 0.778 |  | 0.417 | 0.660 |  | 5.562 | 0.062 |  | 3.508 | 0.173 |
| F3amplitudeLogRelF0_sma3nz_amean | 13.841 | **0.001** |  | 4.154 | 0.125 |  | 2.602 | 0.272 |  | 5.975 | 0.050 |  | 6.236 | 0.044 |  | 4.241 | 0.120 |  | 2.222 | 0.329 |  | 0.076 | 0.963 |  | 7.154 | 0.028 |  | 0.467 | 0.792 |
| F3amplitudeLogRelF0_sma3nz_stddevNorm | 12.372 | **0.002** |  | 2.652 | 0.266 |  | 2.580 | 0.275 |  | 4.828 | 0.089 |  | 8.078 | 0.018 |  | 5.522 | 0.063 |  | 2.374 | 0.305 |  | 0.254 | 0.881 |  | 3.325 | 0.190 |  | 0.790 | 0.674 |
| alphaRatioV_sma3nz_amean | 1.607 | 0.448 |  | 0.182 | 0.913 |  | 3.541 | 0.170 |  | 8.540 | 0.014 |  | 6.105 | 0.047 |  | 6.604 | 0.037 |  | 0.365 | 0.833 |  | 5.134 | 0.077 |  | 7.770 | 0.021 |  | 1.609 | 0.447 |
| alphaRatioV_sma3nz_stddevNorm | 6.200 | 0.045 |  | 3.091 | 0.213 |  | 4.911 | 0.086 |  | 7.169 | 0.028 |  | 5.620 | 0.060 |  | 1.801 | 0.406 |  | 4.031 | 0.133 |  | 5.415 | 0.067 |  | 12.125 | **0.002** |  | 1.643 | 0.440 |
| hammarbergIndexV_sma3nz_amean | 1.694 | 0.429 |  | 0.610 | 0.737 |  | 1.840 | 0.399 |  | 9.922 | 0.007 |  | 5.586 | 0.061 |  | 6.484 | 0.039 |  | 1.845 | 0.398 |  | 5.572 | 0.062 |  | 7.199 | 0.027 |  | 1.167 | 0.558 |
| hammarbergIndexV_sma3nz_stddevNorm | 4.759 | 0.093 |  | 3.928 | 0.140 |  | 4.715 | 0.095 |  | 0.937 | 0.626 |  | 3.634 | 0.162 |  | 0.369 | 0.831 |  | 0.879 | 0.644 |  | 1.519 | 0.468 |  | 12.357 | **0.002** |  | 1.484 | 0.476 |
| slopeV0-500_sma3nz_amean | 21.128 | **<0.001** |  | 4.945 | 0.084 |  | 0.629 | 0.730 |  | 0.505 | 0.777 |  | 2.844 | 0.241 |  | 3.644 | 0.162 |  | 3.126 | 0.209 |  | 5.574 | 0.062 |  | 10.802 | **0.005** |  | 6.588 | 0.037 |
| slopeV0-500_sma3nz_stddevNorm | 5.970 | 0.051 |  | 7.218 | 0.027 |  | 3.171 | 0.205 |  | 0.513 | 0.774 |  | 1.072 | 0.585 |  | 0.148 | 0.929 |  | 2.003 | 0.367 |  | 1.233 | 0.540 |  | 11.799 | **0.003** |  | 0.875 | 0.646 |
| slopeV500-1500_sma3nz_amean | 5.950 | **0.003** |  | 3.154 | 0.046 |  | 1.754 | 0.416 |  | 8.804 | 0.012 |  | 1.690 | 0.430 |  | 9.778 | 0.008 |  | 7.283 | 0.026 |  | 8.109 | 0.000 |  | 9.746 | **<0.001** |  | 0.293 | 0.864 |
| slopeV500-1500_sma3nz_stddevNorm | 14.368 | **0.001** |  | 9.294 | 0.010 |  | 1.509 | 0.470 |  | 13.355 | **0.001** |  | 6.549 | 0.038 |  | 10.513 | 0.005 |  | 5.686 | 0.058 |  | 10.222 | 0.006 |  | 14.409 | **0.001** |  | 0.425 | 0.809 |
| spectralFluxV_sma3nz_amean | 1.428 | 0.490 |  | 0.627 | 0.731 |  | 3.257 | 0.196 |  | 1.770 | 0.413 |  | 3.648 | 0.161 |  | 3.019 | 0.221 |  | 3.367 | 0.186 |  | 2.957 | 0.228 |  | 3.768 | 0.152 |  | 3.019 | 0.221 |
| spectralFluxV_sma3nz_stddevNorm | 0.532 | 0.766 |  | 0.880 | 0.644 |  | 9.927 | 0.007 |  | 1.223 | 0.543 |  | 0.568 | 0.753 |  | 0.404 | 0.817 |  | 2.728 | 0.256 |  | 2.979 | 0.225 |  | 3.004 | 0.223 |  | 0.404 | 0.817 |
| mfcc1V_sma3nz_amean | 1.218 | 0.544 |  | 1.445 | 0.486 |  | 2.415 | 0.299 |  | 7.356 | 0.025 |  | 0.277 | 0.871 |  | 3.880 | 0.144 |  | 0.011 | 0.995 |  | 5.394 | 0.067 |  | 1.726 | 0.422 |  | 3.880 | 0.144 |
| mfcc1V_sma3nz_stddevNorm | 5.548 | 0.062 |  | 2.958 | 0.228 |  | 1.101 | 0.577 |  | 2.843 | 0.241 |  | 3.545 | 0.170 |  | 1.365 | 0.505 |  | 0.109 | 0.947 |  | 0.643 | 0.725 |  | 13.531 | **0.001** |  | 1.365 | 0.505 |
| mfcc2V_sma3nz_amean | 0.179 | 0.836 |  | 1.556 | 0.215 |  | 2.503 | 0.086 |  | 2.415 | 0.093 |  | 0.716 | 0.491 |  | 1.733 | 0.420 |  | 1.192 | 0.307 |  | 5.911 | 0.052 |  | 2.703 | 0.259 |  | 1.733 | 0.420 |
| mfcc2V_sma3nz_stddevNorm | 0.203 | 0.903 |  | 1.023 | 0.600 |  | 1.147 | 0.564 |  | 0.655 | 0.721 |  | 0.611 | 0.737 |  | 0.665 | 0.717 |  | 0.792 | 0.673 |  | 7.905 | 0.019 |  | 0.392 | 0.822 |  | 0.665 | 0.717 |
| mfcc3V_sma3nz_amean | 2.901 | 0.235 |  | 1.592 | 0.451 |  | 3.652 | 0.161 |  | 0.503 | 0.778 |  | 0.173 | 0.917 |  | 3.178 | 0.204 |  | 3.043 | 0.051 |  | 7.659 | 0.022 |  | 6.078 | 0.048 |  | 3.178 | 0.204 |
| mfcc3V_sma3nz_stddevNorm | 0.412 | 0.814 |  | 1.995 | 0.369 |  | 0.213 | 0.899 |  | 1.344 | 0.511 |  | 0.741 | 0.690 |  | 6.276 | 0.043 |  | 1.033 | 0.596 |  | 0.644 | 0.725 |  | 0.381 | 0.826 |  | 6.276 | 0.043 |
| mfcc4V_sma3nz_amean | 5.495 | **0.005** |  | 1.426 | 0.244 |  | 2.803 | 0.246 |  | 1.963 | 0.145 |  | 0.207 | 0.813 |  | 1.674 | 0.433 |  | 0.326 | 0.723 |  | 1.660 | 0.194 |  | 6.119 | 0.047 |  | 1.674 | 0.433 |
| mfcc4V_sma3nz_stddevNorm | 7.089 | 0.029 |  | 5.289 | 0.071 |  | 1.507 | 0.471 |  | 4.010 | 0.135 |  | 11.066 | 0.004 |  | 9.279 | 0.010 |  | 6.081 | 0.048 |  | 3.980 | 0.137 |  | 0.502 | 0.778 |  | 9.279 | 0.010 |
| alphaRatioUV_sma3nz_amean | 1.230 | 0.541 |  | 1.080 | 0.583 |  | 9.424 | 0.009 |  | 1.903 | 0.386 |  | 0.390 | 0.823 |  | 2.054 | 0.358 |  | 3.087 | 0.214 |  | 2.956 | 0.228 |  | 6.022 | 0.049 |  | 2.054 | 0.358 |
| hammarbergIndexUV_sma3nz_amean | 1.505 | 0.471 |  | 1.311 | 0.519 |  | 7.648 | 0.022 |  | 2.116 | 0.347 |  | 0.797 | 0.671 |  | 2.038 | 0.361 |  | 4.276 | 0.118 |  | 1.960 | 0.375 |  | 5.394 | 0.067 |  | 2.038 | 0.361 |
| slopeUV0-500_sma3nz_amean | 0.508 | 0.776 |  | 1.414 | 0.493 |  | 4.325 | 0.115 |  | 2.134 | 0.344 |  | 1.134 | 0.567 |  | 3.428 | 0.180 |  | 3.695 | 0.158 |  | 1.603 | 0.449 |  | 1.134 | 0.567 |  | 3.428 | 0.180 |
| slopeUV500-1500_sma3nz_amean | 4.778 | 0.092 |  | 3.444 | 0.179 |  | 1.661 | 0.436 |  | 2.743 | 0.254 |  | 3.572 | 0.168 |  | 1.339 | 0.512 |  | 8.013 | 0.018 |  | 0.783 | 0.676 |  | 7.064 | 0.029 |  | 1.339 | 0.512 |
| spectralFluxUV_sma3nz_amean | 1.956 | 0.376 |  | 1.812 | 0.404 |  | 7.075 | 0.029 |  | 0.598 | 0.741 |  | 7.092 | 0.029 |  | 4.114 | 0.128 |  | 2.647 | 0.266 |  | 0.448 | 0.799 |  | 2.124 | 0.346 |  | 4.114 | 0.128 |
| loudnessPeaksPerSec | 1.524 | 0.222 |  | 0.830 | 0.439 |  | 0.269 | 0.764 |  | 2.972 | 0.055 |  | 1.383 | 0.254 |  | 2.004 | 0.367 |  | 1.029 | 0.360 |  | 2.510 | 0.085 |  | 5.196 | **0.007** |  | 2.004 | 0.367 |
| VoicedSegmentsPerSec | 0.154 | 0.926 |  | 2.277 | 0.320 |  | 6.406 | 0.041 |  | 2.138 | 0.343 |  | 0.287 | 0.866 |  | 0.796 | 0.672 |  | 2.387 | 0.303 |  | 7.766 | 0.021 |  | 2.257 | 0.324 |  | 0.796 | 0.672 |
| MeanVoicedSegmentLengthSec | 12.443 | **0.002** |  | 5.000 | 0.082 |  | 1.460 | 0.482 |  | 3.679 | 0.159 |  | 3.837 | 0.147 |  | 6.022 | 0.049 |  | 4.370 | 0.112 |  | 2.721 | 0.257 |  | 2.545 | 0.082 |  | 6.022 | 0.049 |
| StddevVoicedSegmentLengthSec | 4.875 | **0.009** |  | 1.678 | 0.191 |  | 0.318 | 0.853 |  | 0.680 | 0.712 |  | 3.965 | 0.021 |  | 4.844 | 0.009 |  | 3.015 | 0.053 |  | 1.245 | 0.537 |  | 1.587 | 0.452 |  | 4.844 | 0.009 |
| MeanUnvoicedSegmentLength | 2.345 | 0.310 |  | 2.132 | 0.344 |  | 6.842 | 0.033 |  | 2.695 | 0.260 |  | 0.532 | 0.767 |  | 0.686 | 0.710 |  | 2.625 | 0.269 |  | 5.329 | 0.070 |  | 7.287 | 0.026 |  | 0.686 | 0.710 |
| StddevUnvoicedSegmentLength | 1.543 | 0.462 |  | 3.026 | 0.220 |  | 14.501 | 0.001 |  | 5.081 | 0.079 |  | 0.135 | 0.935 |  | 1.583 | 0.453 |  | 2.583 | 0.275 |  | 3.198 | 0.202 |  | 4.046 | 0.132 |  | 1.583 | 0.453 |
| equivalentSoundLevel_dBp | 6.331 | 0.042 |  | 3.111 | 0.211 |  | 1.280 | 0.527 |  | 2.237 | 0.327 |  | 6.131 | 0.047 |  | 6.227 | 0.044 |  | 4.233 | 0.120 |  | 1.129 | 0.569 |  | 2.268 | 0.322 |  | 6.227 | 0.044 |
| **Abbreviations**: HR, heart rate; F, frequency; MFCCs, mel-frequency cepstral coefficients; HNR, harmonics-to-noise ratio.  ***Note***: To assess the distinctiveness of groups based on acoustic and physiological features, Shapiro-Wilk tests were first applied for normality assessment. Where normality was confirmed, one-way ANOVA was conducted, reporting F-values; for non-normal distributions, Kruskal-Wallis tests were utilized, with H-values indicated. Bold typeface indicates p-values that remain significant at the 5% level after applying the false discovery rate (FDR) correction. | | | | | | | | | | | | | | | | | | | | | | | | | | | | | |

**Table S5.** The Predictive Performance of the Four Machine Learning Models on the Severe Group for Core Symptoms of SAD (K-SPS, K-LSAS, K-SADS, and K-SIAS) Using the Combination of Grid Search and Stratified Cross-Validation.

| Variable^a^ | | Physiological Features | | | | Acoustic Features | | | | Multimodal Features^b^ | | | |
| --- | --- | --- | --- | --- | --- | --- | --- | --- | --- | --- | --- | --- | --- |
|  |  | K-SPS | K-LSAS | K-SADS | K-SIAS | K-SPS | K-LSAS | K-SADS | K-SIAS | K-SPS | K-LSAS | K-SADS | K-SIAS |
| RF  (Random Forest) | Accuracy | 0.666 | 0.666 | 0.590 | 0.630 | 0.781 | 0.696 | 0.635 | 0.712 | 0.803 | 0.741 | 0.644 | 0.720 |
|  | AUROC | 0.577 | 0.734 | 0.618 | 0.692 | 0.781 | 0.743 | 0.717 | 0.732 | 0.831 | 0.765 | 0.617 | 0.788 |
|  | F1-score | 0.657 | 0.661 | 0.585 | 0.609 | 0.776 | 0.696 | 0.635 | 0.703 | 0.800 | 0.722 | 0.641 | 0.706 |
|  | Sensitivity | 0.666 | 0.666 | 0.590 | 0.630 | 0.781 | 0.696 | 0.635 | 0.712 | 0.803 | 0.741 | 0.644 | 0.720 |
|  | PPV | 0.659 | 0.664 | 0.614 | 0.618 | 0.778 | 0.712 | 0.648 | 0.710 | 0.801 | 0.733 | 0.645 | 0.745 |
|  | NPV | 0.733 | 0.754 | 0.694 | 0.723 | 0.809 | 0.799 | 0.717 | 0.787 | 0.847 | 0.787 | 0.708 | 0.792 |
| XGB  (XGBoost) | Accuracy | 0.605 | 0.689 | 0.546 | 0.607 | 0.728 | 0.742 | 0.689 | 0.606 | 0.712 | 0.765 | 0.668 | 0.674 |
|  | AUROC | 0.556 | 0.702 | 0.615 | 0.591 | 0.767 | 0.799 | 0.732 | 0.630 | 0.742 | **0.843** | 0.702 | 0.720 |
|  | F1-score | 0.603 | 0.684 | 0.537 | 0.599 | 0.722 | 0.740 | 0.688 | 0.609 | 0.710 | 0.760 | 0.656 | 0.674 |
|  | Sensitivity | 0.605 | 0.689 | 0.546 | 0.607 | 0.728 | 0.742 | 0.689 | 0.606 | 0.712 | 0.765 | 0.668 | 0.674 |
|  | PPV | 0.607 | 0.685 | 0.547 | 0.603 | 0.730 | 0.744 | 0.705 | 0.641 | 0.721 | 0.780 | 0.672 | 0.686 |
|  | NPV | 0.689 | 0.758 | 0.648 | 0.708 | 0.783 | 0.809 | 0.767 | 0.746 | 0.797 | 0.836 | 0.716 | 0.774 |
| LGBM  (Light GBM) | Accuracy | 0.674 | 0.643 | 0.576 | 0.623 | 0.727 | 0.711 | 0.727 | 0.674 | 0.758 | 0.728 | 0.742 | 0.689 |
|  | AUROC | 0.626 | 0.647 | 0.605 | 0.637 | 0.772 | 0.762 | 0.746 | 0.665 | 0.811 | 0.818 | 0.799 | 0.735 |
|  | F1-score | 0.661 | 0.639 | 0.570 | 0.615 | 0.722 | 0.712 | 0.728 | 0.665 | 0.753 | 0.730 | 0.738 | 0.685 |
|  | Sensitivity | 0.674 | 0.643 | 0.576 | 0.623 | 0.727 | 0.711 | 0.727 | 0.674 | 0.758 | 0.728 | 0.742 | 0.689 |
|  | PPV | 0.665 | 0.646 | 0.596 | 0.632 | 0.726 | 0.717 | 0.741 | 0.671 | 0.756 | 0.749 | 0.743 | 0.698 |
|  | NPV | 0.721 | 0.736 | 0.691 | 0.742 | 0.783 | 0.788 | 0.786 | 0.751 | 0.805 | 0.815 | 0.779 | 0.779 |
| CAT  (Cat Boost) | Accuracy | 0.652 | 0.667 | 0.575 | 0.691 | 0.766 | 0.726 | 0.712 | 0.644 | 0.796 | 0.720 | 0.750 | 0.720 |
|  | AUROC | 0.567 | 0.754 | 0.600 | 0.711 | 0.773 | 0.779 | 0.795 | 0.721 | **0.852** | 0.810 | **0.810** | **0.795** |
|  | F1-score | 0.645 | 0.665 | 0.555 | 0.665 | 0.763 | 0.719 | 0.712 | 0.624 | 0.791 | 0.717 | 0.746 | 0.709 |
|  | Sensitivity | 0.652 | 0.667 | 0.575 | 0.691 | 0.766 | 0.726 | 0.712 | 0.644 | 0.796 | 0.720 | 0.750 | 0.720 |
|  | PPV | 0.650 | 0.675 | 0.569 | 0.672 | 0.764 | 0.726 | 0.723 | 0.617 | 0.796 | 0.742 | 0.760 | 0.727 |
|  | NPV | 0.726 | 0.757 | 0.660 | 0.786 | 0.811 | 0.777 | 0.778 | 0.730 | 0.833 | 0.809 | 0.802 | 0.803 |
| **Abbreviations**: SAD, social anxiety disorder; K-SPS, the Korean version of the social phobia scale; K-LSAS, the Korean version of the liebowitz social anxiety scale; K-SADS, the Korean version of the social avoidance and distress scale; K-SIAS, the Korean version of the social interaction anxiety scale; AUROC, area under the receiver operating characteristic; PPV, positive predictive value; NPV, negative predictive value.  ***Note***: ^a^ The highest AUROC scores for each clinical and psychological scale are highlighted in bold to denote superior model performance.  ^b^ The combination of physiological and acoustic features. | | | | | | | | | | | | | |

**Table S6.** The Predictive Performance of the Four Machine Learning Models on the Severe Group for Cognitive Symptoms of SAD (PERS, BFNE, and ISS) Using the Combination of Grid Search and Stratified Cross-Validation.

| Variable^a^ | | Physiological Features | | | Acoustic Features | | | Multimodal Features^b^ | | |
| --- | --- | --- | --- | --- | --- | --- | --- | --- | --- | --- |
|  |  | PERS | BFNE | ISS | PERS | BFNE | ISS | PERS | BFNE | ISS |
| RF  (Random Forest) | Accuracy | 0.689 | 0.423 | 0.584 | 0.635 | 0.682 | 0.651 | 0.726 | 0.636 | 0.652 |
|  | AUROC | 0.733 | 0.380 | 0.580 | 0.645 | 0.748 | 0.655 | 0.772 | 0.722 | 0.626 |
|  | F1-score | 0.686 | 0.423 | 0.585 | 0.627 | 0.680 | 0.641 | 0.720 | 0.636 | 0.641 |
|  | Sensitivity | 0.689 | 0.423 | 0.584 | 0.635 | 0.682 | 0.651 | 0.726 | 0.636 | 0.652 |
|  | PPV | 0.687 | 0.431 | 0.587 | 0.627 | 0.700 | 0.642 | 0.728 | 0.656 | 0.639 |
|  | NPV | 0.762 | 0.512 | 0.681 | 0.715 | 0.779 | 0.712 | 0.805 | 0.736 | 0.708 |
| XGB  (XGBoost) | Accuracy | 0.674 | 0.553 | 0.637 | 0.727 | 0.628 | 0.614 | 0.712 | 0.651 | 0.584 |
|  | AUROC | 0.655 | 0.512 | 0.593 | 0.737 | 0.709 | 0.624 | 0.777 | 0.728 | 0.648 |
|  | F1-score | 0.672 | 0.556 | 0.638 | 0.727 | 0.625 | 0.608 | 0.711 | 0.646 | 0.586 |
|  | Sensitivity | 0.674 | 0.553 | 0.637 | 0.727 | 0.628 | 0.614 | 0.712 | 0.651 | 0.584 |
|  | PPV | 0.676 | 0.565 | 0.646 | 0.734 | 0.634 | 0.605 | 0.720 | 0.657 | 0.592 |
|  | NPV | 0.754 | 0.638 | 0.724 | 0.812 | 0.690 | 0.701 | 0.790 | 0.691 | 0.687 |
| LGBM  (Light GBM) | Accuracy | 0.651 | 0.425 | 0.599 | 0.651 | 0.629 | 0.674 | 0.757 | 0.667 | 0.674 |
|  | AUROC | 0.666 | 0.437 | 0.606 | 0.685 | 0.675 | 0.750 | 0.847 | 0.678 | 0.749 |
|  | F1-score | 0.653 | 0.420 | 0.597 | 0.645 | 0.631 | 0.660 | 0.756 | 0.668 | 0.673 |
|  | Sensitivity | 0.651 | 0.425 | 0.599 | 0.651 | 0.629 | 0.674 | 0.757 | 0.667 | 0.674 |
|  | PPV | 0.661 | 0.459 | 0.606 | 0.650 | 0.650 | 0.660 | 0.762 | 0.693 | 0.674 |
|  | NPV | 0.741 | 0.532 | 0.692 | 0.747 | 0.732 | 0.738 | 0.817 | 0.779 | 0.746 |
| CAT  (Cat Boost) | Accuracy | 0.674 | 0.462 | 0.591 | 0.727 | 0.651 | 0.689 | 0.779 | 0.735 | 0.742 |
|  | AUROC | 0.694 | 0.439 | 0.566 | 0.780 | 0.738 | 0.732 | **0.862** | **0.775** | **0.765** |
|  | F1-score | 0.673 | 0.459 | 0.594 | 0.720 | 0.653 | 0.685 | 0.782 | 0.737 | 0.740 |
|  | Sensitivity | 0.674 | 0.462 | 0.591 | 0.727 | 0.651 | 0.689 | 0.779 | 0.735 | 0.742 |
|  | PPV | 0.677 | 0.496 | 0.600 | 0.728 | 0.660 | 0.686 | 0.798 | 0.756 | 0.746 |
|  | NPV | 0.754 | 0.584 | 0.695 | 0.799 | 0.727 | 0.753 | 0.876 | 0.837 | 0.807 |
| **Abbreviations**: SAD, social anxiety disorder; PERS, the post-event rumination scale; BFNE, the brief fear of negative evaluation; ISS, the internalized shame scale; AUROC, area under the receiver operating characteristic; PPV, positive predictive value; NPV, negative predictive value.  ***Note***: ^a^ The highest AUROC scores for each clinical and psychological scale are highlighted in bold to denote superior model performance.  ^b^ The combination of physiological and acoustic features. | | | | | | | | | | |

**Table S7.** The Predictive Performance of the Four Machine Learning Models on the Severe Group for Generalized Anxiety (STAI-State, STAI-Trait, and BAI) Using the Combination of Grid Search and Stratified Cross-Validation.

| Variable^a^ | | Physiological Features | | | Acoustic Features | | | Multimodal Features^b^ | | |
| --- | --- | --- | --- | --- | --- | --- | --- | --- | --- | --- |
|  |  | STAI-State | STAI-Trait | BAI | STAI-State | STAI-Trait | BAI | STAI-State | STAI-Trait | BAI |
| RF  (Random Forest) | Accuracy | 0.585 | 0.591 | 0.552 | 0.560 | 0.629 | 0.668 | 0.644 | 0.720 | 0.706 |
|  | AUROC | 0.652 | 0.655 | 0.512 | 0.562 | 0.629 | 0.720 | 0.685 | 0.755 | 0.781 |
|  | F1-score | 0.585 | 0.594 | 0.549 | 0.550 | 0.628 | 0.667 | 0.641 | 0.720 | 0.703 |
|  | Sensitivity | 0.585 | 0.591 | 0.552 | 0.560 | 0.629 | 0.668 | 0.644 | 0.720 | 0.706 |
|  | PPV | 0.620 | 0.611 | 0.561 | 0.557 | 0.636 | 0.687 | 0.652 | 0.733 | 0.714 |
|  | NPV | 0.716 | 0.699 | 0.653 | 0.653 | 0.709 | 0.741 | 0.709 | 0.791 | 0.783 |
| XGB  (XGBoost) | Accuracy | 0.555 | 0.577 | 0.553 | 0.629 | 0.621 | 0.675 | 0.630 | 0.727 | 0.651 |
|  | AUROC | 0.623 | 0.599 | 0.549 | 0.690 | 0.621 | 0.705 | 0.693 | 0.744 | 0.741 |
|  | F1-score | 0.557 | 0.576 | 0.543 | 0.620 | 0.623 | 0.670 | 0.627 | 0.726 | 0.652 |
|  | Sensitivity | 0.555 | 0.577 | 0.553 | 0.629 | 0.621 | 0.675 | 0.630 | 0.727 | 0.651 |
|  | PPV | 0.592 | 0.584 | 0.562 | 0.657 | 0.628 | 0.676 | 0.644 | 0.734 | 0.656 |
|  | NPV | 0.688 | 0.671 | 0.669 | 0.722 | 0.707 | 0.732 | 0.708 | 0.798 | 0.738 |
| LGBM  (Light GBM) | Accuracy | 0.562 | 0.561 | 0.468 | 0.660 | 0.673 | 0.743 | 0.630 | 0.766 | 0.741 |
|  | AUROC | 0.599 | 0.625 | 0.518 | 0.719 | 0.673 | 0.751 | 0.701 | **0.819** | 0.765 |
|  | F1-score | 0.565 | 0.556 | 0.471 | 0.656 | 0.667 | 0.739 | 0.626 | 0.766 | 0.736 |
|  | Sensitivity | 0.562 | 0.561 | 0.468 | 0.660 | 0.673 | 0.743 | 0.630 | 0.766 | 0.741 |
|  | PPV | 0.571 | 0.585 | 0.495 | 0.667 | 0.691 | 0.753 | 0.650 | 0.776 | 0.746 |
|  | NPV | 0.649 | 0.676 | 0.604 | 0.729 | 0.734 | 0.788 | 0.718 | 0.827 | 0.807 |
| CAT  (Cat Boost) | Accuracy | 0.538 | 0.607 | 0.478 | 0.689 | 0.720 | 0.698 | 0.682 | 0.750 | 0.728 |
|  | AUROC | 0.598 | 0.619 | 0.497 | 0.716 | 0.720 | 0.770 | **0.740** | 0.796 | **0.797** |
|  | F1-score | 0.539 | 0.604 | 0.480 | 0.687 | 0.720 | 0.692 | 0.681 | 0.751 | 0.728 |
|  | Sensitivity | 0.538 | 0.607 | 0.478 | 0.689 | 0.720 | 0.698 | 0.682 | 0.750 | 0.728 |
|  | PPV | 0.562 | 0.620 | 0.492 | 0.689 | 0.725 | 0.704 | 0.696 | 0.760 | 0.728 |
|  | NPV | 0.650 | 0.712 | 0.607 | 0.741 | 0.792 | 0.746 | 0.745 | 0.817 | 0.788 |
| **Abbreviations**: SAD, social anxiety disorder; STAI-State, the state-trait anxiety inventory-state; STAI-Trait, the state-trait anxiety inventory-trait; BAI, the beck anxiety inventory; AUROC, area under the receiver operating characteristic; PPV, positive predictive value; NPV, negative predictive value.  ***Note***: ^a^ The highest AUROC scores for each clinical and psychological scale are highlighted in bold to denote superior model performance.  ^b^ The combination of physiological and acoustic features. | | | | | | | | | | |

**Table S8.** The Predictive Performance of the Four Machine Learning Models on the Severe Group for Core Symptoms of SAD (K-SPS, K-LSAS, K-SADS, and K-SIAS) Using the Combination of Random Search and Stratified Cross-Validation.

| Variable^a^ | | Physiological Features | | | | Acoustic Features | | | | Multimodal Features^b^ | | | |
| --- | --- | --- | --- | --- | --- | --- | --- | --- | --- | --- | --- | --- | --- |
|  |  | K-SPS | K-LSAS | K-SADS | K-SIAS | K-SPS | K-LSAS | K-SADS | K-SIAS | K-SPS | K-LSAS | K-SADS | K-SIAS |
| RF  (Random Forest) | Accuracy | 0.651 | 0.643 | 0.530 | 0.652 | 0.766 | 0.665 | 0.636 | 0.667 | 0.788 | 0.741 | 0.644 | 0.705 |
|  | AUROC | 0.576 | 0.711 | 0.604 | 0.702 | 0.783 | 0.691 | 0.732 | 0.736 | 0.830 | 0.772 | 0.697 | 0.783 |
|  | F1-score | 0.635 | 0.633 | 0.526 | 0.627 | 0.762 | 0.665 | 0.635 | 0.660 | 0.784 | 0.713 | 0.642 | 0.695 |
|  | Sensitivity | 0.651 | 0.643 | 0.530 | 0.652 | 0.766 | 0.665 | 0.636 | 0.667 | 0.788 | 0.741 | 0.644 | 0.705 |
|  | PPV | 0.642 | 0.635 | 0.560 | 0.706 | 0.765 | 0.683 | 0.644 | 0.669 | 0.783 | 0.727 | 0.658 | 0.707 |
|  | NPV | 0.719 | 0.738 | 0.661 | 0.726 | 0.805 | 0.772 | 0.714 | 0.771 | 0.829 | 0.767 | 0.736 | 0.787 |
| XGB  (XGBoost) | Accuracy | 0.651 | 0.689 | 0.576 | 0.585 | 0.743 | 0.758 | 0.697 | 0.598 | 0.697 | 0.731 | 0.691 | 0.674 |
|  | AUROC | 0.576 | 0.713 | 0.607 | 0.603 | 0.746 | 0.794 | 0.741 | 0.623 | 0.736 | 0.742 | 0.709 | 0.721 |
|  | F1-score | 0.635 | 0.684 | 0.568 | 0.580 | 0.738 | 0.756 | 0.699 | 0.602 | 0.693 | 0.750 | 0.680 | 0.674 |
|  | Sensitivity | 0.651 | 0.689 | 0.576 | 0.585 | 0.743 | 0.758 | 0.697 | 0.598 | 0.697 | 0.772 | 0.691 | 0.674 |
|  | PPV | 0.643 | 0.684 | 0.580 | 0.591 | 0.742 | 0.761 | 0.711 | 0.636 | 0.701 | 0.833 | 0.697 | 0.686 |
|  | NPV | 0.712 | 0.757 | 0.678 | 0.697 | 0.803 | 0.819 | 0.774 | 0.747 | 0.775 | 0.731 | 0.734 | 0.774 |
| LGBM  (Light GBM) | Accuracy | 0.674 | 0.650 | 0.546 | 0.630 | 0.727 | 0.719 | 0.735 | 0.644 | 0.758 | 0.736 | 0.787 | 0.660 |
|  | AUROC | 0.626 | 0.651 | 0.578 | 0.626 | 0.788 | 0.759 | 0.754 | 0.669 | 0.806 | **0.820** | 0.800 | 0.711 |
|  | F1-score | 0.658 | 0.647 | 0.542 | 0.623 | 0.724 | 0.719 | 0.734 | 0.636 | 0.751 | 0.737 | 0.783 | 0.646 |
|  | Sensitivity | 0.674 | 0.650 | 0.546 | 0.630 | 0.727 | 0.719 | 0.735 | 0.644 | 0.758 | 0.736 | 0.787 | 0.660 |
|  | PPV | 0.669 | 0.655 | 0.552 | 0.634 | 0.726 | 0.725 | 0.745 | 0.640 | 0.757 | 0.754 | 0.790 | 0.652 |
|  | NPV | 0.717 | 0.744 | 0.652 | 0.737 | 0.789 | 0.790 | 0.773 | 0.726 | 0.799 | 0.818 | 0.816 | 0.729 |
| CAT  (Cat Boost) | Accuracy | 0.652 | 0.682 | 0.561 | 0.683 | 0.735 | 0.750 | 0.696 | 0.659 | 0.804 | 0.728 | 0.750 | 0.713 |
|  | AUROC | 0.562 | 0.723 | 0.608 | 0.712 | 0.782 | 0.777 | 0.762 | 0.724 | **0.850** | 0.819 | **0.822** | **0.808** |
|  | F1-score | 0.647 | 0.677 | 0.547 | 0.660 | 0.730 | 0.746 | 0.694 | 0.649 | 0.802 | 0.727 | 0.748 | 0.707 |
|  | Sensitivity | 0.652 | 0.682 | 0.561 | 0.683 | 0.735 | 0.750 | 0.696 | 0.659 | 0.804 | 0.728 | 0.750 | 0.713 |
|  | PPV | 0.655 | 0.684 | 0.558 | 0.670 | 0.729 | 0.753 | 0.717 | 0.664 | 0.809 | 0.747 | 0.758 | 0.719 |
|  | NPV | 0.722 | 0.755 | 0.664 | 0.787 | 0.785 | 0.802 | 0.781 | 0.760 | 0.856 | 0.817 | 0.792 | 0.804 |
| **Abbreviations**: SAD, social anxiety disorder; K-SPS, the Korean version of the social phobia scale; K-LSAS, the Korean version of the liebowitz social anxiety scale; K-SADS, the Korean version of the social avoidance and distress scale; K-SIAS, the Korean version of the social interaction anxiety scale; AUROC, area under the receiver operating characteristic; PPV, positive predictive value; NPV, negative predictive value.  ***Note***: ^a^ The highest AUROC scores for each clinical and psychological scale are highlighted in bold to denote superior model performance.  ^b^ The combination of physiological and acoustic features. | | | | | | | | | | | | | |

**Table S9.** The Predictive Performance of the Four Machine Learning Models on the Severe Group for Cognitive Symptoms of SAD (PERS, BFNE, and ISS) Using the Combination of Random Search and Stratified Cross-Validation.

| Variable^a^ | | Physiological Features | | | Acoustic Features | | | Multimodal Features^b^ | | |
| --- | --- | --- | --- | --- | --- | --- | --- | --- | --- | --- |
|  |  | PERS | BFNE | ISS | PERS | BFNE | ISS | PERS | BFNE | ISS |
| RF  (Random Forest) | Accuracy | 0.689 | 0.446 | 0.614 | 0.643 | 0.690 | 0.696 | 0.750 | 0.599 | 0.644 |
|  | AUROC | 0.744 | 0.397 | 0.600 | 0.653 | 0.758 | 0.669 | 0.749 | 0.665 | 0.629 |
|  | F1-score | 0.687 | 0.444 | 0.614 | 0.632 | 0.687 | 0.688 | 0.744 | 0.590 | 0.631 |
|  | Sensitivity | 0.689 | 0.446 | 0.614 | 0.643 | 0.690 | 0.696 | 0.750 | 0.599 | 0.644 |
|  | PPV | 0.688 | 0.448 | 0.622 | 0.635 | 0.698 | 0.691 | 0.754 | 0.595 | 0.628 |
|  | NPV | 0.759 | 0.535 | 0.702 | 0.719 | 0.760 | 0.744 | 0.806 | 0.679 | 0.700 |
| XGB  (XGBoost) | Accuracy | 0.666 | 0.523 | 0.576 | 0.635 | 0.628 | 0.569 | 0.689 | 0.651 | 0.538 |
|  | AUROC | 0.643 | 0.502 | 0.560 | 0.675 | 0.718 | 0.615 | 0.774 | 0.732 | 0.633 |
|  | F1-score | 0.665 | 0.524 | 0.579 | 0.639 | 0.622 | 0.563 | 0.689 | 0.644 | 0.539 |
|  | Sensitivity | 0.666 | 0.523 | 0.576 | 0.635 | 0.628 | 0.569 | 0.689 | 0.651 | 0.538 |
|  | PPV | 0.669 | 0.532 | 0.590 | 0.650 | 0.634 | 0.563 | 0.708 | 0.655 | 0.545 |
|  | NPV | 0.754 | 0.606 | 0.688 | 0.740 | 0.679 | 0.667 | 0.788 | 0.688 | 0.653 |
| LGBM  (Light GBM) | Accuracy | 0.659 | 0.432 | 0.607 | 0.764 | 0.644 | 0.652 | 0.773 | 0.667 | 0.674 |
|  | AUROC | 0.665 | 0.443 | 0.589 | 0.787 | 0.687 | 0.740 | 0.864 | 0.694 | **0.758** |
|  | F1-score | 0.660 | 0.415 | 0.608 | 0.762 | 0.642 | 0.644 | 0.772 | 0.668 | 0.673 |
|  | Sensitivity | 0.659 | 0.432 | 0.607 | 0.764 | 0.644 | 0.652 | 0.773 | 0.667 | 0.674 |
|  | PPV | 0.666 | 0.482 | 0.619 | 0.772 | 0.684 | 0.644 | 0.777 | 0.700 | 0.674 |
|  | NPV | 0.746 | 0.559 | 0.703 | 0.840 | 0.779 | 0.735 | 0.835 | 0.791 | 0.746 |
| CAT  (Cat Boost) | Accuracy | 0.659 | 0.523 | 0.591 | 0.750 | 0.652 | 0.689 | 0.787 | 0.705 | 0.719 |
|  | AUROC | 0.683 | 0.472 | 0.567 | 0.823 | 0.734 | 0.733 | **0.866** | **0.778** | 0.748 |
|  | F1-score | 0.662 | 0.522 | 0.591 | 0.751 | 0.652 | 0.690 | 0.785 | 0.707 | 0.717 |
|  | Sensitivity | 0.659 | 0.523 | 0.591 | 0.750 | 0.652 | 0.689 | 0.787 | 0.705 | 0.719 |
|  | PPV | 0.669 | 0.526 | 0.595 | 0.762 | 0.659 | 0.694 | 0.788 | 0.727 | 0.719 |
|  | NPV | 0.755 | 0.610 | 0.692 | 0.832 | 0.727 | 0.773 | 0.843 | 0.807 | 0.784 |
| **Abbreviations**: SAD, social anxiety disorder; PERS, the post-event rumination scale; BFNE, the brief fear of negative evaluation; ISS, the internalized shame scale; AUROC, area under the receiver operating characteristic; PPV, positive predictive value; NPV, negative predictive value.  ***Note***: ^a^ The highest AUROC scores for each clinical and psychological scale are highlighted in bold to denote superior model performance.  ^b^ The combination of physiological and acoustic features. | | | | | | | | | | |

**Table S10.** The Predictive Performance of the Four Machine Learning Models on the Severe Group for Generalized Anxiety (STAI-State, STAI-Trait, and BAI) Using the Combination of Random Search and Stratified Cross-Validation.

| Variable^a^ | | Physiological Features | | | Acoustic Features | | | Multimodal Features^b^ | | |
| --- | --- | --- | --- | --- | --- | --- | --- | --- | --- | --- |
|  |  | STAI-State | STAI-Trait | BAI | STAI-State | STAI-Trait | BAI | STAI-State | STAI-Trait | BAI |
| RF  (Random Forest) | Accuracy | 0.577 | 0.592 | 0.582 | 0.590 | 0.621 | 0.668 | 0.621 | 0.720 | 0.705 |
|  | AUROC | 0.652 | 0.671 | 0.514 | 0.584 | 0.718 | 0.734 | 0.662 | 0.772 | 0.786 |
|  | F1-score | 0.574 | 0.596 | 0.575 | 0.589 | 0.621 | 0.666 | 0.618 | 0.719 | 0.695 |
|  | Sensitivity | 0.577 | 0.592 | 0.582 | 0.590 | 0.621 | 0.668 | 0.621 | 0.720 | 0.705 |
|  | PPV | 0.621 | 0.615 | 0.599 | 0.592 | 0.631 | 0.688 | 0.623 | 0.730 | 0.702 |
|  | NPV | 0.720 | 0.702 | 0.672 | 0.674 | 0.704 | 0.741 | 0.692 | 0.795 | 0.763 |
| XGB  (XGBoost) | Accuracy | 0.554 | 0.569 | 0.553 | 0.629 | 0.628 | 0.721 | 0.630 | 0.704 | 0.689 |
|  | AUROC | 0.609 | 0.628 | 0.535 | 0.681 | 0.674 | 0.735 | 0.679 | 0.731 | 0.743 |
|  | F1-score | 0.557 | 0.568 | 0.543 | 0.620 | 0.631 | 0.713 | 0.623 | 0.704 | 0.690 |
|  | Sensitivity | 0.554 | 0.569 | 0.553 | 0.629 | 0.628 | 0.721 | 0.630 | 0.704 | 0.689 |
|  | PPV | 0.587 | 0.583 | 0.561 | 0.649 | 0.642 | 0.719 | 0.650 | 0.718 | 0.691 |
|  | NPV | 0.682 | 0.670 | 0.668 | 0.718 | 0.725 | 0.763 | 0.720 | 0.791 | 0.764 |
| LGBM  (Light GBM) | Accuracy | 0.501 | 0.562 | 0.476 | 0.660 | 0.673 | 0.713 | 0.683 | 0.727 | 0.704 |
|  | AUROC | 0.565 | 0.588 | 0.530 | 0.689 | 0.708 | 0.773 | **0.732** | **0.795** | 0.758 |
|  | F1-score | 0.500 | 0.565 | 0.482 | 0.657 | 0.668 | 0.702 | 0.679 | 0.725 | 0.695 |
|  | Sensitivity | 0.501 | 0.562 | 0.476 | 0.660 | 0.673 | 0.713 | 0.683 | 0.727 | 0.704 |
|  | PPV | 0.526 | 0.584 | 0.505 | 0.665 | 0.691 | 0.729 | 0.696 | 0.732 | 0.701 |
|  | NPV | 0.606 | 0.681 | 0.613 | 0.722 | 0.736 | 0.769 | 0.755 | 0.789 | 0.752 |
| CAT  (Cat Boost) | Accuracy | 0.546 | 0.615 | 0.523 | 0.637 | 0.704 | 0.713 | 0.689 | 0.742 | 0.719 |
|  | AUROC | 0.581 | 0.624 | 0.547 | 0.694 | 0.754 | 0.740 | 0.729 | 0.793 | **0.809** |
|  | F1-score | 0.546 | 0.615 | 0.516 | 0.634 | 0.701 | 0.709 | 0.689 | 0.742 | 0.721 |
|  | Sensitivity | 0.546 | 0.615 | 0.523 | 0.637 | 0.704 | 0.713 | 0.689 | 0.742 | 0.719 |
|  | PPV | 0.580 | 0.627 | 0.523 | 0.645 | 0.711 | 0.713 | 0.709 | 0.755 | 0.724 |
|  | NPV | 0.675 | 0.719 | 0.631 | 0.719 | 0.765 | 0.763 | 0.762 | 0.821 | 0.794 |
| **Abbreviations**: SAD, social anxiety disorder; STAI-State, the state-trait anxiety inventory-state; STAI-Trait, the state-trait anxiety inventory-trait; BAI, the beck anxiety inventory; AUROC, area under the receiver operating characteristic; PPV, positive predictive value; NPV, negative predictive value.  ***Note***: ^a^ The highest AUROC scores for each clinical and psychological scale are highlighted in bold to denote superior model performance.  ^b^ The combination of physiological and acoustic features. | | | | | | | | | | |
